# An integrated transcriptional analysis of the developing human retina

**DOI:** 10.1242/dev.169474

**Published:** 2019-01-29

**Authors:** Carla B. Mellough, Roman Bauer, Joseph Collin, Birthe Dorgau, Darin Zerti, David W. P. Dolan, Carl M. Jones, Osagie G. Izuogu, Min Yu, Dean Hallam, Jannetta S. Steyn, Kathryn White, David H. Steel, Mauro Santibanez-Koref, David J. Elliott, Michael S. Jackson, Susan Lindsay, Sushma Grellscheid, Majlinda Lako

**Affiliations:** 1 Institute of Genetic Medicine, Newcastle University, Newcastle NE1 3BZ, UK; 2 Lions Eye Institute, 2 Verdun Street, Nedlands, Perth, WA 6009, Australia; 3 School of Computing, Newcastle University, Newcastle NE4 5TG, UK; 4 Department of Biosciences, Durham University, Stockton Road, Durham DH1 3LE, UK; 5 European Bioinformatics Institute (EMBL-EBI), Wellcome Genome Campus, Cambridge CB10 1SD, UK; 6 EM Research Services, Newcastle University, Newcastle NE2 4HH, UK

**Keywords:** Developing human retina, Transcriptome, Immunohistochemistry, Alternative splicing, Retinal cells, Circular RNAs

## Abstract

The scarcity of embryonic/foetal material as a resource for direct study means that there is still limited understanding of human retina development. Here, we present an integrated transcriptome analysis combined with immunohistochemistry in human eye and retinal samples from 4 to 19 post-conception weeks. This analysis reveals three developmental windows with specific gene expression patterns that informed the sequential emergence of retinal cell types and enabled identification of stage-specific cellular and biological processes, and transcriptional regulators. Each stage is characterised by a specific set of alternatively spliced transcripts that code for proteins involved in the formation of the photoreceptor connecting cilium, pre-mRNA splicing and epigenetic modifiers. Importantly, our data show that the transition from foetal to adult retina is characterised by a large increase in the percentage of mutually exclusive exons that code for proteins involved in photoreceptor maintenance. The circular RNA population is also defined and shown to increase during retinal development. Collectively, these data increase our understanding of human retinal development and the pre-mRNA splicing process, and help to identify new candidate disease genes.

## Abstract (precis)

**Highlighted Article:** An integrated transcriptional and immunohistochemical analysis of human retinogenesis *in vivo* that is essential for the identification of master genes that cause retinal disease and for the development of innovative retinal therapies.

## INTRODUCTION

The development of the human eye and the visual system is a complex process that has attracted great interest throughout history (Heavner and Pevny, 2012; Reeves and Taylor, 2004). Human eye development begins at post conception week (PCW) 3.5 and continues until the 5th postnatal month. It begins with the expression of a set of transcription factors (TFs) within the neuroectodermal plate known as the eye field (Zuber et al., 2003). Signalling from the pre-chordal mesoderm splits the single eye field, which then forms two optic vesicles around the 4th week of human development. The optic vesicles undergo complex patterning and morphogenesis, with the distal optic vesicle developing into neural retina with cell fate decisions following the order of retinal histogenesis. The vertebrate retina is composed of five neuronal and one glial cell type (Müller glia) that are organized within three different layers. Each of the retinal cell types is generated in an orderly manner that has been well studied in vertebrates (Marquardt and Gruss, 2002).

This process has been more difficult to document in humans as development occurs over many months both *in utero* and postnatally; however, advances made in optical coherence tomography and the availability of a limited number of human embryonic and foetal samples have enabled visualization of retinal layers as well as some immunohistochemical (IHC) and molecular studies over the course of human gestation (Vajzovic et al., 2012; Aldiri et al., 2017; Hoshino et al., 2017; Hendrickson, 2016; Nag and Wadhwa, 2006-07; Provis et al., 1985; Cornish et al., 2004; Hendrickson and Zhang, 2017; O'Brien et al., 2003; Hendrickson et al., 2008; Hendrickson et al., 2012). This protracted window of retinal development and limited availability of human developmental retinal samples has meant that most of the molecular and functional data to date are obtained from model organisms (Quan et al., 2012; Zuber, 2010; Edqvist et al., 2006; Furukawa et al., 1997), which are unable to fully replicate human disease phenotypes due to anatomical, genetic and functional species-specific differences (Hodges et al., 2002; Bibb et al., 2001; Baker, 2013; Seabrook et al., 2017). In this work, we have undertaken an integrated transcriptomic and IHC study of human eye histogenesis with a focus on the neural retina up to 19 PCW, in order to expand our knowledge of human development and provide human data for use in the research and clinical ophthalmic community. Furthermore, we have performed a systematic splicing analysis during human retinal development and have identified splice variants that are involved in the formation and function of the photoreceptor connecting cilium, the splicing process itself and epigenetic modifications.

## RESULTS

### Transcriptome dynamics of the developing human eye and retina define three key developmental windows

We performed RNA-seq studies of 21 samples obtained from embryonic and foetal human retinae (7.7-18 PCW) and compared them with three samples of adult human retinae. RNA was also obtained from eight whole embryonic eyes (4.6-8 PCW) and subjected to RNA-seq analysis. In total, 32 strand-specific RNA-seq datasets with 65-92% of uniquely mapped reads per sample were obtained (Table S1).

**Fig. 1.**
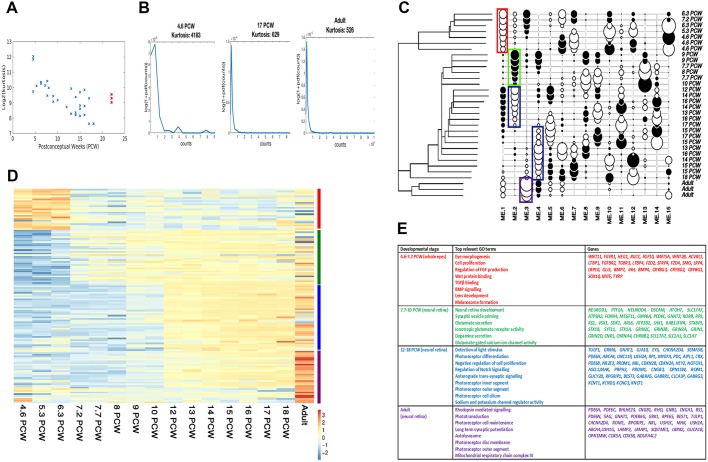
**RNA-seq analysis defines three developmental windows characterised by stage-specific transcriptional expression.** (A,B) Kurtosis analysis showing a decrease as development proceeds; the adult retina samples are shown in red (A). (C) ME-based cluster analysis with developmental windows highlighted in red (4.6-7.2 PCW), green (7.7-10 PCW), blue (12-18 PCW) and purple (adult retina); the scores for the 15 different MEs are shown in black (positive) or white (negative), with circle size proportional to the absolute value. (D) Z scores of upregulated genes for each stage of development defined by ME-based cluster analysis (comparisons performed between two sequential stages, e.g.4.6-7.2 PCW versus 7.7-10 PCW). Blue and red colours in the heat map correspond to low and high gene expression, respectively, and bars on the right-hand side indicate the developmental stages (red for 4.6-7.2 PCW, green for 7.7-10 PCW, blue for 12-18 PCW and purple for adult retina). (E) GO analysis of upregulated genes for each stage of development defined by ME-based cluster analysis (comparisons performed between two sequential stages, e.g. 4.6-7.2 PCW versus 7.7-10 PCW).

After quality control and normalisation of the data, we investigated how the overall expression of protein-coding genes changed during development. To achieve this, we characterized the distribution of protein-coding gene expression at each developmental stage by computing the kurtosis. The kurtosis quantifies how heavy-tailed or light-tailed a distribution is compared with a normal distribution, i.e. higher kurtosis is associated with the presence of extreme values. Fig. 1A shows that the kurtosis significantly decreased as the developmental stage increased [correlation coefficient between log_2_(kurtosis) and developmental stage is R=−0.77, *P*<0.0001], indicating that at early stages of retinal development the expression profile was less balanced and more heterogeneous than at later stages. Fig. 1B visualizes representative gene expression distributions from early (left), late (middle) developmental and adult (right) stages. Late stages were characterised by a smoother distribution than early stages, showing that retinal gene expression converged to a more stable profile as development progressed.

To identify stage-specific gene expression patterns, we adapted a multi-component approach using Moran Eigen-vectors (MEs). Individual samples were first clustered using hierarchical clustering. Afterwards, they were aligned with respect to 15 inferred ME components, as described in the Materials and methods section. Of these components, the first four MEs ordered samples in an unsupervised way, reminiscent of development: ME1 low, developing eyes from 4.6-7.2 PCW; ME2 high, developing retinae from 7.7-10 PCW; ME2 and ME4 low, developing retinae from 12-18 PCW; and ME3 low, adult retinae (Fig. 1C,D). Differential expression analysis between neighbouring groups was carried out (Table S2), enabling identification of a large number of transcripts whose expression changed during early human eye development. Gene ontology analysis (GO) of transcripts significantly altered during 4.6-7.2 PCW indicated enrichment of genes implicated in eye morphogenesis, mesenchymal to epithelial transition and cellular proliferation, as well as those acting in key pathways already shown to play an important role in both eye and neural retinal genesis of various animal models, such as fibroblast growth factor, WNT, TGFβ/BMP and SHH signalling (Fig. 1E). A significant upregulation of genes involved in lens and melanosome development was observed, suggesting the emergence of pigmented cells and lens during this stage of development. These transcriptional data were supported by our IHC analysis, which showed the presence of bilateral developing optic cups on each side of the developing forebrain at 4 PCW (Fig. S1A-C). These underwent invagination to give rise to RPE and the developing neural retina which, at 5.7 PCW, appears as a single neuroblastic layer with loosely radially aligned nestin immunoreactive neural progenitor cells (Fig. S1D-F,G). By 6.3 PCW, the lens had separated from the surface epithelium and was internalised within the optic cup, with the lens fibres showing clear expression of crystalline αB (CRYAB) (Fig. S2A), lens germinal epithelium showing expression of insulin-like growth factor 1 receptor (IGF1R) (Fig. S2A), and lens fibres and anterior lens epithelium showing nuclear SOX1 (arrows) expression (Fig. S2B,C). As the development of the neural retina proceeds, radial nestin expression becomes more prominent towards the basal aspect of the neuroepithelium by 7.4 PCW (Fig. S1I,K). At 7.8 PCW, an inner neuroblastic zone (INBZ) and outer neuroblastic zone (ONBZ) are now visible. Over the ensuing weeks of development, the neuroblastic layers continue to thicken and neural and retinal markers, including VSX2, OTX2, SOX2 and PAX6 start to emerge. Interestingly, VSX2, which plays a major role in eye organogenesis (Zou and Levine, 2012), can be observed at the peripheral margin of the developing retina as early as 6.5 PCW (Fig. S1H), but was not detected in the central retina until 7.8 PCW (Fig. S1L). Strong nuclear SOX2 expression is also observed at the ONBZ at 7.8 PCW (Fig. S1O). OTX2, a protein with multiple roles in the retina, including RPE, photoreceptor and bipolar cell specification and maintenance (Beby and Lamonerie, 2013), was detected at 8 PCW across the developing retina, with a greater concentration of OTX2-immunopositive nuclei in the ONBZ (Fig. S1T,U). Expression of PAX6, which has a known role in regulating the multipotency of retinal progenitors (Marquardt et al., 2001; Mathers and Jamrich, 2000), was first observed in the INBZ at 7.8 PCW (Fig. S1N), then in the developing retinal ganglion cell layer (dGCL) at 8 PCW (Fig. S1Q), corroborating data reported by Hendrickson (2016). Surprisingly, the central neural retina was not immunoreactive at early stages of development (<7.8 PCW) for many of the classically understood early eye field markers (e.g. OTX2; Fig. S1J).

Transition to the second stage of development (7.7-10 PCW) was associated with high expression of a large number of retinal progenitor cell (RPC) markers (Fig. S3A) and upregulation of genes involved in neural retinal development, central nervous system maturation, synaptogenesis, glutamate secretion and receptor signalling, dopamine secretion, and neuronal action potential propagation (Table S2 and Fig. 1E). During this developmental window, expression of key genes reported to play important roles in the emergence of retinal cells types was noted. For example, cone-specific transducin (*GNAT2*), RAR-related orphan receptor B (*RORB*) and cGMP-specific 3′,5'-cyclic phosphodiesterase subunit alpha (*PDE6C*) were significantly upregulated alongside genes regulating RGC (*ATOH7* and *NEUROD1*), and horizontal cell (*FOXN4*) and amacrine cell (*CABP1* and *EPHA8*) development. Together, these transcriptional data support the idea that during 7.7-10 PCW, the transcriptional programmes that underlie the emergence of RGCs, horizontal, amacrine and cone photoreceptors are initiated. These findings were underlined by iRegulon analysis (Table S3), which predicted transcription factors such as *REST* (which is important in regulating RGC gene expression in retinal progenitor cells; Mao et al., 2011), *TEAD4* [part of a pathway that controls the switch between retinal progenitor cell proliferation and photoreceptor differentiation (Asaoka et al., 2014) as well as RPE development (Miesfeld et al., 2015)] and *POU4F3* (an inducer of RGC development; Brown, 2011) to be the main regulators that were differentially expressed during the transition from 4.6-7.2 PCW to 7.7-10 PCW.

GO analysis indicated that, during 12-18 PCW, processes related to phototransduction, photoreceptor cell differentiation and the light response were highly prominent (Table S2 and Fig. 1E) in addition to negative regulation of cellular proliferation, which indicates the switch from the proliferative to the differentiation stage of photoreceptor development. This was supported by IHC analysis showing Ki67 staining in the outer neuroblastic zone at 8 PCW, which spread to inner neuroblastic zone and ganglion cell layer as development proceeded (Fig. S3B). During this developmental window, we noticed upregulation of genes such as *EYES* and *PRDM1*, which have been shown to be required for the correct function and survival of photoreceptors, as well as maintaining photoreceptor cell identity by repressing alternative pathways. We also observed upregulation of transcription factors that activate rod development (*NR2E3* and *NRL*) as well as genes that are involved in phototransduction in rods (*CNGB1* and *GNAT1*), suggesting initiation of the transcriptional programmes that underlie rod emergence and function. Importantly, cellular processes related to development of the photoreceptor inner segment, connecting cilium and outer segment were highly evident (Table S2 and Fig. 1E), thus suggesting further photoreceptor structural development during this period of foetal development, which was underlined by our TEM analysis (Fig. S4A-C). This was further supported by the iRegulon analysis (Table S3), which identified known regulators of photoreceptor cell fates (*OTX2*, *ONECUT1*, *EP300*, *CRX*, *RAX2*, *RXRA*, *CTCF*, *NRL* and *NR2E3*) (Furukawa et al., 1997; Nishida et al., 2003; Hennig et al., 2013; Mo et al., 2016) and ciliogenesis (*RFX1*). Initiation of gene expression related to phototransduction was also accompanied by a significant upregulation of genes involved in scavenger receptor activity (Table S2), which act to clean up and regenerate the metabolic by-products arising from highly metabolic tissue, such as the retina, which has a unique photo-oxidative environment and photo-transduction processes.

The process of phototransduction and photoreceptor cell differentiation continues throughout the developmental periods studied, as observed by the GO analysis of genes that significantly changed their expression between adult versus foetal retina (Table S2 and Fig. 1E). A specific feature of this developmental transition is the upregulation of genes involved in rhodopsin-mediated signalling, which suggests that the development of rods continues beyond the last developmental stage included in our RNA-seq study (18 PCW). Other specific features identified by this analysis include the development of photoreceptor membrane discs within photoreceptor outer segments to enable the phototransduction process which were clearly observed by TEM (Fig. S4D-F), genes involved in the clearance of protein aggregates and dysfunctional organelles through the macroautophagy-lysosomal pathway, as well as those implicated in the mitochondrial respiratory chain complex IV*.* It is of interest to note that iRegulon also identified *RAB7A* (Table S3), a key player in the maturation of autophagosomes and endosomes (Hyttinen et al., 2013) together with transcription factors controlling cell apoptosis (*JUNB*) and senescence (*CBX7*), consistent with the onset of programmed cell death in the retina, which is shown to occur from the 13-18 PCW in the inner nuclear layer (INL) and 21-23 PCW in the outer nuclear layer (ONL) (Vecino et al., 2004)*.*

**Fig. 2.**
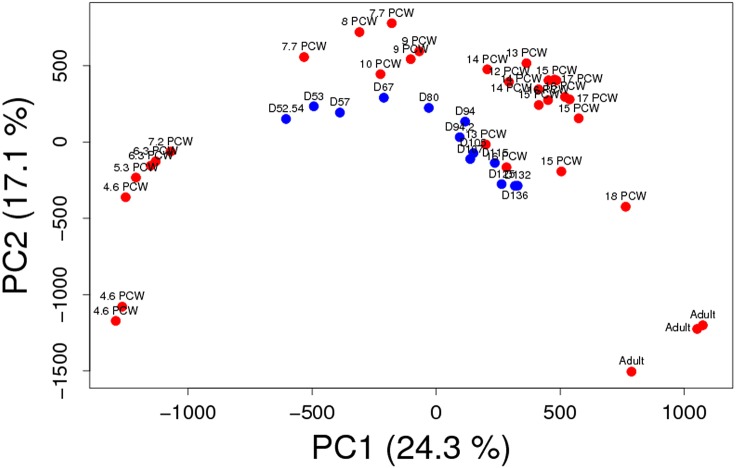
**PCA analysis and heatmap data.** Comparison of the RNA-seq samples analysed in this study (red) versus these reported by Hoshino et al. (2017) (blue). Both datasets display a consistent pattern of developmental progression in accordance with one another. Note also that the spread of the datasets mirrors the extent of their developmental time span.

Collectively, the RNA-seq data gathered from our study defines three key developmental windows that are characterised by specific transcriptomic profiles that follow the predicted sequence of eye and retinal histogenesis, and constitute a valuable resource for staging of pluripotent stem cell-derived retinal organoids. While our study was in the final stages of preparation, RNA-seq and IHC data of central and peripheral retina was published by another group (Hoshino et al., 2017). We used our pipeline analysis and compared the samples reported by Hoshino et al. (2017) with ours to evaluate whether this would enhance our current results and conclusions (Fig. 2). This analysis revealed a succinct overlap between the day 52-136 samples reported by Hoshino et al. (2017) and our 7.7-17 PCW, all of which fell within two developmental windows (or epochs) that encompass day 52-67/7.7-10 PCW and day 80-136/12-17 PCW, respectively. Importantly, principal component (PCA) analysis demonstrated a clear sequential progression in both cases (Fig. 2).

### The development of retinal cell types

**Fig. 3.**
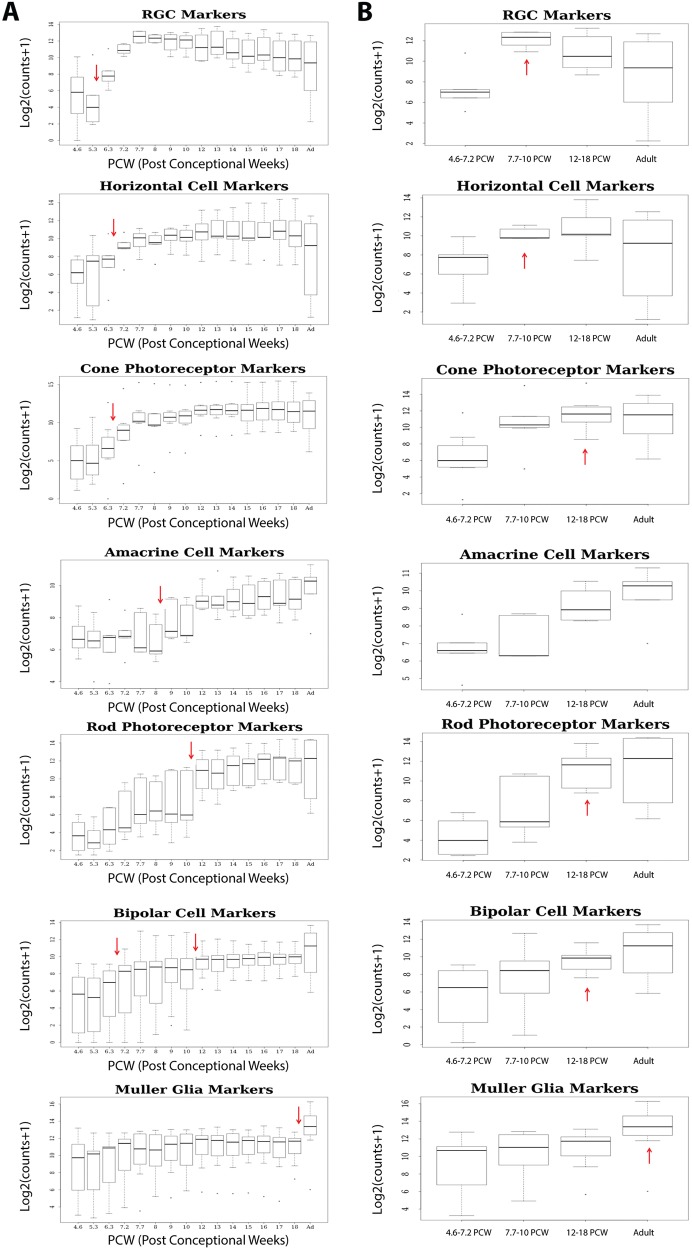
**The expression of retinal marker genes.** (A) Expression of retinal marker genes plotted as log2 transformed counts at each developmental stage included in the RNA-seq analysis. The Wilcoxon rank-sum test was performed on the expression differences between developmental stages to identify the earliest stage with a significant and sustained increase in the expression of retinal lineage cell markers. Ad, adult retina. (B) Expression of retinal markers across the three developmental windows (defined by ME-based cluster analysis) and adult retina plotted as log2 transformed counts per million. The Wilcoxon rank-sum test was performed on the expression differences to identify developmental stages with peak expression of these retinal markers. The red arrows indicate a significant and sustained increase in expression (*P*<0.05). (A,B) The line that divides the box shows the median, while the box indicates the upper and lower quartiles. Whiskers represent the highest and lowest value excluding outliers, while dots show outlier values outside 1.5 times the interquartile range above the upper quartile and below the lower quartile.

An important feature of cell differentiation in the vertebrate retina is the chronological sequence in which the retinal cell types are generated. RGCs and horizontal cells differentiate first, followed by cone photoreceptors, amacrine cells, rods, bipolar cells and Müller glial cells (Marquardt and Gruss, 2002). To assess whether such chronological sequence could be mimicked by the transcriptomic profiles, we computed and plotted the expression of key markers (shown in Fig. S5), which have been shown in various key publications (Bassett and Wallace, 2012; Marquardt, 2003; Ohsawa and Kageyama, 2008; Swaroop et al., 2010; Andreazzoli, 2009; Poché and Reese, 2009; Reese, 2011; Macosko et al., 2015) to direct the specification of each retinal cell type. The Wilcoxon rank-sum test was performed on the expression differences between developmental stages to identify the earliest stage with a significant and sustained increase in the expression of retinal lineage cell-specific markers (Fig. 3A). In parallel, expression of these markers with regards to the three developmental windows defined in the previous section was also calculated (Fig. 3B) to assess peak marker expression.

**Fig. 4.**
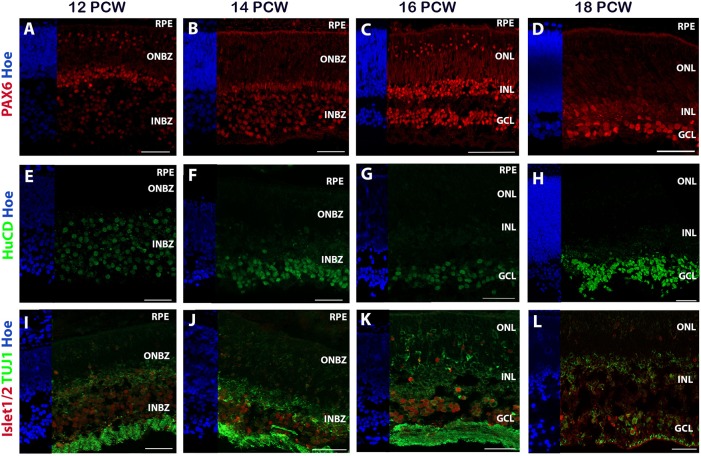
**Immunohistochemical analysis of developing human retina at 12-18 PCW.** (A-D) PAX6 and (E-L) the retinal ganglion cell markers HuC/D, islet 1/2 and TUJ1in the human foetal retina at 12-18 PCW. INBZ, inner neuroblastic zone; ONBZ, outer neuroblastic zone; RPE, retinal pigment epithelium. Scale bars: 50 µm.

This analysis indicated that the markers that characterise RGCs showed a pronounced upregulation from 5.3 PCW (Fig. 3A), although their peak expression was noted at 7.7-10 PCW (Fig. 3B). To investigate how the transcriptional data correlated with protein expression, we examined the developmental expression profile of RGCs by IHC (Fig. 4). Developing RGCs were observed at the basal aspect of the INBZ, the location of the future ganglion cell layer (GCL), as early as 8 PCW (Fig. S1Q). The eye-field marker PAX6 is also required for RGC transcriptional activation (Riesenberg et al., 2009) and its expression was observed in both ONBZ and INBZ at 12 and 14 PCW (Fig. 4A,B). At 16 PCW, its expression was observed in the basal side of the INL and GCL, and in a few cells with elongated nuclei in the ONL (Fig. 4C). PAX6 expression became restricted to the basal side of INL and GCL at 18 PCW (Fig. 4D). HuC/D, a marker of RGCs and amacrine cells, was expressed in the INBZ at 12 and 14 PCW (Fig. 4E,F), and in the developing GCL at 16 and 18 PCW (Fig. 4G,H). Low expression of the neuron-specific class III β-tubulin (TUJ1) could be observed across the developing retinal neuroepithelium at 6.5 PCW and 7.8 PCW (Fig. S1G,L), and by 12 PCW this was localised to the basal side of the ONBZ and strongly in the developing nerve fibre layer (Fig. 4I). As the development proceeds, TUJ1 expression is observed in some RGCs and their processes which extended across the inner surface of the retina in perfect alignment, travelling towards the optic nerve head and forming a rudimentary nerve fibre layer (Fig. 4J,K). This expression pattern was further confirmed by double immunostaining with TUJ1 and islet1/2 antibodies (islet1/2 is a marker of developing RGCs, bipolar and amacrine cells; Elshatory et al., 2007), which can be clearly observed at 16 and 18 PCW (Fig. 4K,L). Current reports have provided contrasting data on RGC development with these being reported as early as 4 PCW by Nag and Wadhwa (2006-07) and 8 PCW by Hendrickson (2016). Our data corroborate the latter and show that the first observable developing RGCs are evident at 8 PCW and the RGC processes are well established at 18 PCW.

Horizontal cell markers showed a significant upregulation from 6.3 PCW, reaching peak expression at 7.7-10 PCW, which was maintained throughout foetal development and adulthood (Fig. 3A,B). This expression profile fits nicely with the first reported emergence of immature horizontal cells at day 59 of human development (Hoshino et al., 2017). IHC analysis of calbindin D28k, a marker for horizontal, cone bipolar, wide-field amacrine and large retinal ganglion cells was first observed at 10 PCW in the INBZ (Fig. S6A). At 16 PCW, strong expression was observed in the GCL and weaker expression in the two band-like patterns in the developing INL (presumably where amacrine and horizontal cells will develop) and ONL (where cone bipolars are likely developing) (Fig. S6B, arrows). At 18 PCW, strong expression in the GCL and weaker expression at the basal side of the INL was maintained; however, brighter expression at the apical edge of ONL and at the border between ONL and INL, where the OPL will develop, was observed, suggesting the well-established presence of horizontal cells (Fig. S6C, arrows).

**Fig. 5.**
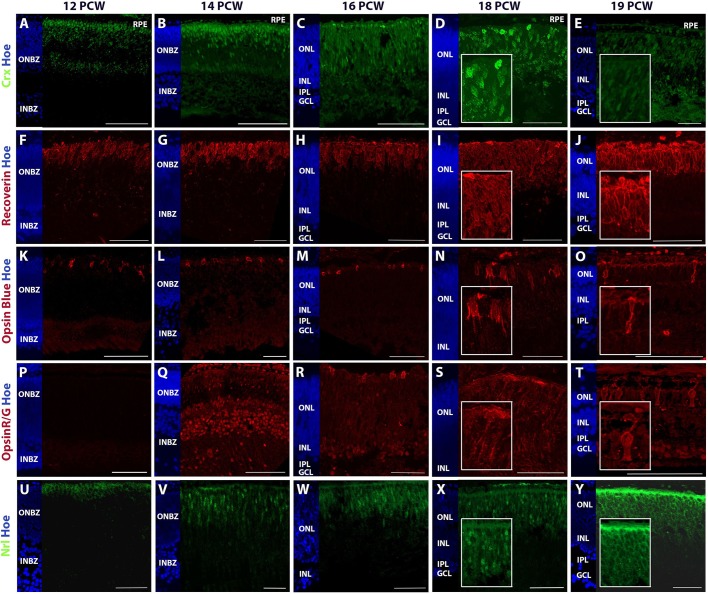
**Analysis of photoreceptor marker expression in the human foetal retina at 12-19 PCW.** The emergence of developing and subtypes of photoreceptors were determined by detection with photoreceptor precursor marker CRX (A-E), pan photoreceptor marker recoverin (F-J), cone photoreceptor markers (K-O) opsin blue and (P-T) opsin red/green, and (U-Y) the rod photoreceptor precursor marker NRL. Hoechst staining is in blue. Scale bars: 100 µm in B,C,K,L,M,O,Q,T,V,X; 50 µm in A,D,F,G-J,N,P,R,S,W; 20 µm in Y. High-magnification insets at 18 and 19 PCW are added to show photoreceptor morphology.

**Fig. 6.**
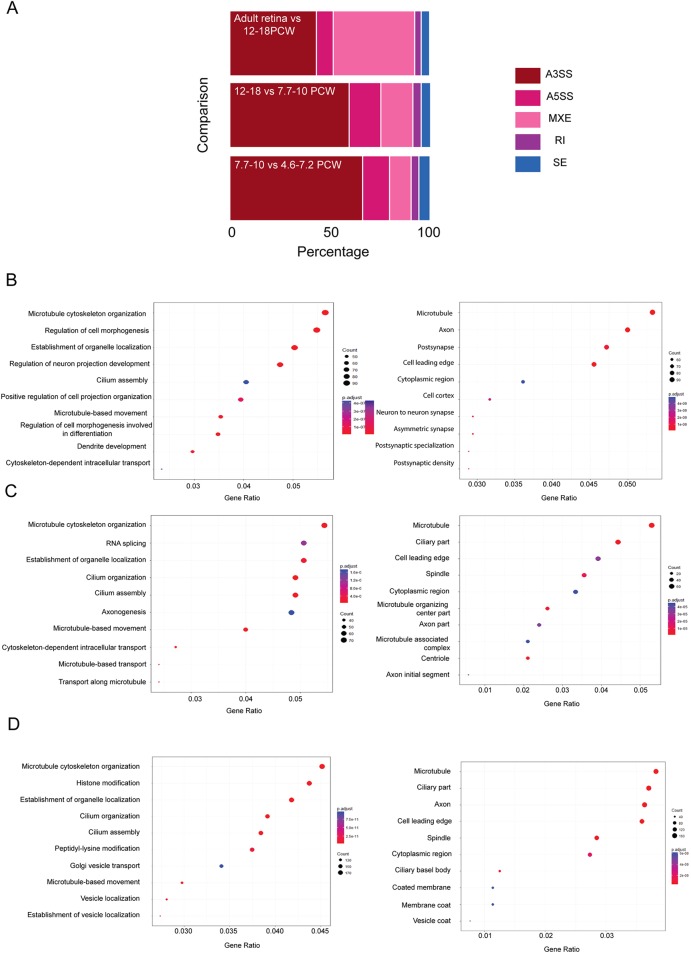
**Stage-specific pre-mRNA splicing during human retinal development.** (A) rMATS analysis showing the percentage of transcripts containing retained introns (RI), skipped exons (SE), alternative 3′ splice sites (A3SS), alternative 5′ splice sites (A5SS) and mutually exclusive exons (MXE). (B-D) Gene ontology enrichment analysis showing biological (left-hand panels) and cellular (right-hand panels) processes affected by alternative splicing during human retinal development. (B) 7.7-10 versus 4.6-7.2 PCW; (C) 12-18 versus 7.7-10 PCW; (D) adult retina versus 12-18 PCW.

Cone precursor cell markers show a distinct increase from 6.3 PCW (Fig. 3A), reaching peak expression at 12-18 PCW, which was comparable with the expression observed in adulthood (Fig. 3B). Nuclear expression of the post-mitotic photoreceptor marker cone-rod homeobox gene (CRX) was detected by IHC at 12-14 PCW, starting as a band of immunopositive nuclei positioned at the apical edge of the ONL at 12 PCW, which then spanned the entire ONL at 16 PCW (Fig. 5A-C). By 18 PCW, large CRX-positive nuclei lined the apical edge of the ONL once more, with paler, smaller nuclei observed throughout the ONL (Fig. 5D). At 19 PCW, pale CRX-immunopositive nuclei were also observed in the INL in addition to the ONL (Fig. 5E). The pan-photoreceptor marker recoverin was clearly observed by 12 PCW, revealing an apical band of developing photoreceptors that thickened over time to 18 PCW (Fig. 5F-I) and then started to recede in depth (Fig. 5J), corresponding with the observation of more mature photoreceptor morphology, as detected by antibodies directed against mature photoreceptor markers: the opsins. The cone photoreceptor marker opsin blue (OPN1SW) was evident in small morphologically immature cone photoreceptors at the apical edge of the neural retina as early as 12 PCW (Fig. 5K), with cells beginning to develop clear photoreceptor-like morphology by 19 PCW (Fig. 5L-O), corroborating published reports (O'Brien et al., 2003; Hendrickson et al., 2008). Opsin red/green (OPN1LW/OPN1MW) showed specific expression to developing cone photoreceptors in the ONL at 16 PCW (Fig. 6R), with clear photoreceptor-like morphology emerging by 19 PCW (Fig. 5T), supporting data published by Hendrickson (2016). The transcriptional analysis performed on individual opsin genes indicated that the short wave length cone opsin (*OPN1SW*) was upregulated from 8 PCW and the medium (*OPN1MW*) and long (*OPN1LW*) wave length opsins were upregulated from 15 and 17 PCW, respectively (data not shown). These results suggest transcriptional activation of key markers well in advance of their IHC detection.

Amacrine cell markers first showed upregulation from 8 PCW (Fig. 3A); nonetheless, there were no significant changes in expression between the three developmental windows or adult retina (Fig. 3B). The IHC analysis indicated that AP2α-positive cells were first detected from 14 PCW (Fig. S6D) with strong nuclear staining in a few cells in the INBZ (arrows) and more abundantly in the basal aspect of the ONBZ. At 16 PCW, only a few immunopositive cells are observed in the GCL and developing ONL (Fig. S6E, arrows); however, cells with clear and strong nuclear expression of this marker are found in the basal side of the INL, suggesting the emergence of amacrine cells (Fig. S6E). At 18 PCW, strong expression of AP2α at the basal aspect of the INL is maintained and at the same time clearer expression is observed in cells residing in the border zone between ONL and INL, suggesting the established presence of horizontal cells (Fig. S6F, arrows), supporting the data obtained with calbindin D28k (Fig. S6C).

In agreement with observations made in the developing retina (Nag and Wadhwa, 2006-07), rod precursor markers were upregulated from 10 PCW, reaching peak expression over 12-18 PCW (Fig. 3B), which was comparable with the expression observed in adulthood, suggesting that rod precursor emergence is subsequent to cone precursors emergence. Immunohistochemically, NRL expression was observed at a similar developmental time to the CRX, with nuclear expression throughout the ONL at 12 and 14 PCW as first reported by Hendrickson and colleagues (O'Brien et al., 2003; Hendrickson et al., 2008), and more clearly visible NRL-positive cells forming an apical band of nuclei in the developing ONL at 16-18 PCW, followed by the loss of nuclear NRL expression at 19 PCW (Fig. 5U-Y). At 19 PCW, NRL immunoreactivity was most evident at the apical edge of the retina, at the border between the RPE and the developing inner and outer segments, as well as the rod soma (Fig. 5Y). The expression of rhodopsin (validated by two different antibodies: RetP1 and Rho) is absent at 18 PCW; however, it is clearly visible at 19 PCW in a similar pattern to NRL (Fig. S7A,B, Fig. 5X,Y), albeit with weaker intensity when compared with adult retina (Fig. S7D,E). The expression of Gαt1, a marker of rod outer segments, was observed throughout the retina with slightly more prominent expression at the apical edge of the ONL at 19 PCW (Fig. S7C); albeit much weaker than the expression observed in adult tissue where immunopositive rod outer segments are clearly visible (Fig. S7F). Together, these data suggest that although rod precursors may be observed as early as 12 PCW, maturing rod photoreceptor morphology is most likely present from 19 PCW, corroborating published findings (Hendrickson et al., 2008, 2012).

Bipolar cell markers showed significant upregulation from 6.3 and 10 PCW (Fig. 3A), which matches the first emergence of cone and rod precursor markers, and may suggest the initiation of transcriptional machinery that underlies the emergence of cone and rod bipolar cells. However, peak expression was observed at 12-18 PCW and was comparable with the expression observed at adulthood (Fig. 3B). Immunohistochemically, the expression of PKCα, a marker for rod bipolar cells, was first observed in the GCL, IPL and basal aspect of the INL (Fig. S6G) at 18 PCW. At 19 PCW, punctate expression of PKCα in the IPL was maintained, and stronger expression around cell bodies emerged in the border between ONL and INL (Fig. S6H, arrows). Clear expression of VSX2 was also observed in the INL, suggesting the emergence of bipolar cells (Fig. S6I), in agreement with recently published data showing mature bipolar expression (CABP5) after day 110 of development (Hoshino et al., 2017).

The markers characterising Müller glial cells showed significant upregulation only when the last foetal stage was compared with adulthood, where peak expression was also noted (Fig. 3A,B). For the IHC analysis, we used antibodies against CRALBP, a marker for Müller glia and RPE cells. This analysis indicated that CRALB was expressed in the RPE from 6.3 PCW (data not shown). In the neural retina, weak CRALBP expression was detected across the neural retina from 12 PCW, showing strongest expression in the INBZ and apical edge of the ONBZ (Fig. S6J). At 16 PCW, immunostaining was observed in the GCL with weaker expression in the developing ONL and INL (Fig. S6K). At 18 PCW, a typical immunostaining pattern of Müller glia cells running from the apical to the basal edges of the neural retina was established with prominent Müller glia end feet observed at the presumptive developing ILM (Fig. S6L).

Resident microglia have been described as the immunological watchdogs of the retina and have been found in foetal retina from early in development (around 8 PCW). Their integration into developing human retina occurs via two main sources: the retinal margin and the optic disc (Diaz-Araya et al., 1995). Our RNA-seq data indicated a significant upregulation of microglia markers during transition from 17 to 18 PCW (Fig. S8A). The expression of these markers was also significantly increased when the foetal stages were compared with adulthood (Fig. S8B). Together, these data may suggest a later than previously reported invasion of microglia in the developing retina, which starts around 17 PCW and continues throughout the rest of foetal development until adulthood. Astrocytes also enter the retina from the brain along the developing optic nerve. Astrocyte precursors are first detected at the edge of the retina by 16 PCW (Chu et al., 2001). In accordance with this, we observed a significant increase in the expression of astrocyte markers only when the last foetal stage (18 PCW) was compared with adult retina (Fig. S8A,B).

Collectively, our transcriptomic data inform the chronological emergence of retinal cell types during human development and support the sequential activation of transcriptional machinery that underlies the development of RGCs, horizontal cells, cone photoreceptors, amacrine cells, rod photoreceptors and, finally, bipolar and Müller glial cells, which appear last (Fig. S9). The comparison of our transcriptomic and IHC data suggests that, for the majority of the markers, the peak mRNA expression matches the earliest detection by IHC. Both of these are preceded by the first significant upregulation in marker gene expression that most likely signifies the activation of lineage-specific transcriptional machinery in advance of the emergence of retinal cell types.

### Stage-specific roles for pre-mRNA splicing during retinal development revealed by alternative splicing analysis

Alternative splicing is a pre-mRNA processing step regulating the selection of specific exons/introns to produce different transcripts from one genomic locus (Kelemen et al., 2013; Wahl et al., 2009). Retinal tissue has one of the highest levels of alternative splicing, and mutations in splicing factors and dysregulation of splicing are associated with retinal diseases (Liu and Zack, 2013). A recent study in mouse has shown that retinal development is characterised by dynamic changes in splicing, with differential splicing events occurring more frequently during early development (Wan et al., 2011). In particular, the photoreceptors are characterised by a specific splicing program that displays a switch-like pattern with high exon inclusion levels in photoreceptors and almost complete exclusion outside the retina (Murphy et al., 2016).

To assess the role of pre-mRNA splicing during human retinal development, we identified target transcripts characterised by skipped exons, retained introns, alternative 5′ and 3′ splice sites, and mutually exclusive exons (Table S4) using rMATS. This analysis (Fig. 6A) revealed that the highest percentage of transcripts containing skipped exons was observed when 7.7-10 PCW samples were compared with the earliest development stages (4.6-7.2 PCW samples). The percentage of transcripts with retained introns as well as those with mutually exclusive exons was very slightly increased when 12-18 PCW were compared with 7.7-10 PCW samples. In contrast, the percentage of transcripts with mutually exclusive exons was significantly increased when adult human retinae were compared with 12-18 PCW samples. GO enrichment analysis indicated that the transcripts with mutually exclusive exons which were significantly increased during the foetal to adult transition coded for proteins involved in photoreceptor maintenance (data not shown).

**Fig. 7.**
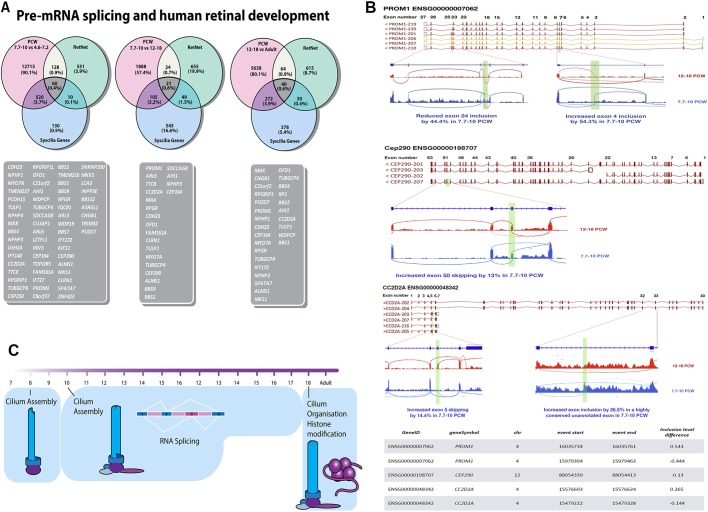
**Alternatively spliced transcripts include genes associated with inherited retinal disease and ciliogenesis.** (A) Cross comparison of alternatively spliced transcripts identified during retinal development with genes associated with retinal disease (retnet) and cilia genes (syscilia). (B) Examples of three genes (*PROM1*, *CEP290* and *CC2D2A*) regulated via alternative splicing between 12-18 and 7.7-10 PCW. The splicing events are illustrated using IGV sashimi plots (see Table S4 for a full list). Transcript numbers are Ensembl identifiers. Green highlights indicate alternative splicing events. (C) Schematic representation of key processes affected by pre-mRNA splicing during human retinal development from 7.7 PCW to adult.

GO enrichment analysis for biological and cellular components (Fig. 6B) identified alternatively spliced transcripts coding proteins involved in connecting cilium assembly, microtubule formation, axon and synapse formation (Fig. 6B and Table S5) during the transition from 4.4-7.2 to 7.7-10 PCW. Among the alternatively spliced transcripts, a large proportion of genes connected with cilium formation was observed, in line with data obtained in murine adult retina showing pre-mRNA splicing to affect genes involved in cilia formation (Murphy et al., 2016). Cell cycle and centrosomal proteins were present in the ‘cilium organisation and assembly’ category revealed by GO enrichment analysis, which may suggest that some of the proteins involved in RPC proliferation during early development may be reused for the cilium assembly and organisation at the later stages. 17.4% of the alternatively spliced transcripts during this developmental window were also differentially expressed and these coded for proteins involved in melanosome and axon formation, voltage-gated sodium channel complexes and synapse formation (data not shown). A cross comparison of alternatively spliced transcripts during this developmental window to retinal disease-associated genes (sph.uth.edu/retnet/) revealed 188 common genes (Fig. 7A), which were associated primarily with photoreceptor cell maintenance, connecting cilia and photoreceptor cell differentiation*.* Sixty of these common 188 alternatively spliced transcripts were involved in cilia formation (Fig. 7A), corroborating recent data linking impaired alternative splicing to cilia genes and inherited retinal dystrophies (Parfitt et al., 2016).

GO enrichment analysis for the transition from 7.7-10 PCW to 12-18 PCW (Fig. 6C) identified alternatively spliced transcripts involved in RNA splicing itself (Table S5), which may indicate the setup of a specific splicing programme during this developmental window. Alternatively, spliced transcripts coding for proteins involved in connecting cilium, axon formation, focal adhesion, cell-substrate adherens junctions and nuclear specks were also detected. We performed the same analysis on the RNA-seq data deposited by Hoshino et al. (2017) for the corresponding stages (day 80-136 versus day 52-67) revealed by our comparative PCA analysis (Fig. 3). Top enriched cellular and biological pathways matched perfectly between the two studies, thus validating our analysis and highlighting the pre-mRNA splicing to be among the top enriched pathways affected by the splicing process during this developmental transition (Fig. S10). 9.1% of the alternatively spliced genes were also differentially expressed and these coded for proteins involved in the formation of photoreceptor outer segments and connecting cilia (data not shown). Cross comparison of alternatively spliced transcripts to retinal disease and ciliary genes identified 21 common genes that are involved in cilia formation and are shown to change in their splicing pattern during the transition from 7.7-10 to 12-18 PCW (Fig. 7A). A detailed description of splicing events for three of these genes is shown in Fig. 7B.

Transition from foetal to adult retina was associated with alternative splicing of genes coding histone modification proteins as well as those involved in cilia, Golgi vesicle transport and axon formation (Table S5 and Fig. 6D). An important role for epigenetic modification of histone 3 has been highlighted for maturation of a subset of bipolar cells (Watanabe and Murakami, 2016). Histone methylation and acetylation has also been shown to regulate RGC development and survival, while histone acetylation and deacetylation have been implicated in photoreceptor cell fate specification and terminal differentiation (Rao et al., 2011). Our data suggest that setting of this specific epigenetic programme may be influenced by alternative splicing of genes coding histone modification proteins. Cross comparison of alternatively spliced transcripts identified during this developmental window to retinal disease and cilia-associated genes identified 40 common genes, as shown in Fig. 7A. A larger proportion (36.5%) of the differentially spliced transcripts were differentially expressed during the transition from foetal to adult retina when compared with 4.6-18 PCW, thus indicating an increasing role for pre-mRNA splicing in regulation of gene expression. The transcripts that were both differentially spliced and expressed, coded for genes involved in chromatin regulation, in photoreceptor inner and outer segment formation, and in connecting cilia and axon formation (data not shown). Together, our data highlight an important role for pre-mRNA splicing during human retinal development and suggest that this may affect important cellular processes, including the assembly and organisation of connecting cilia, establishment of a retinal-specific splicing programme and epigenetic modifications at distinct stages of retinal development (Fig. 7C).

### Increased circular RNA abundance during human retinal development

**Fig. 8.**
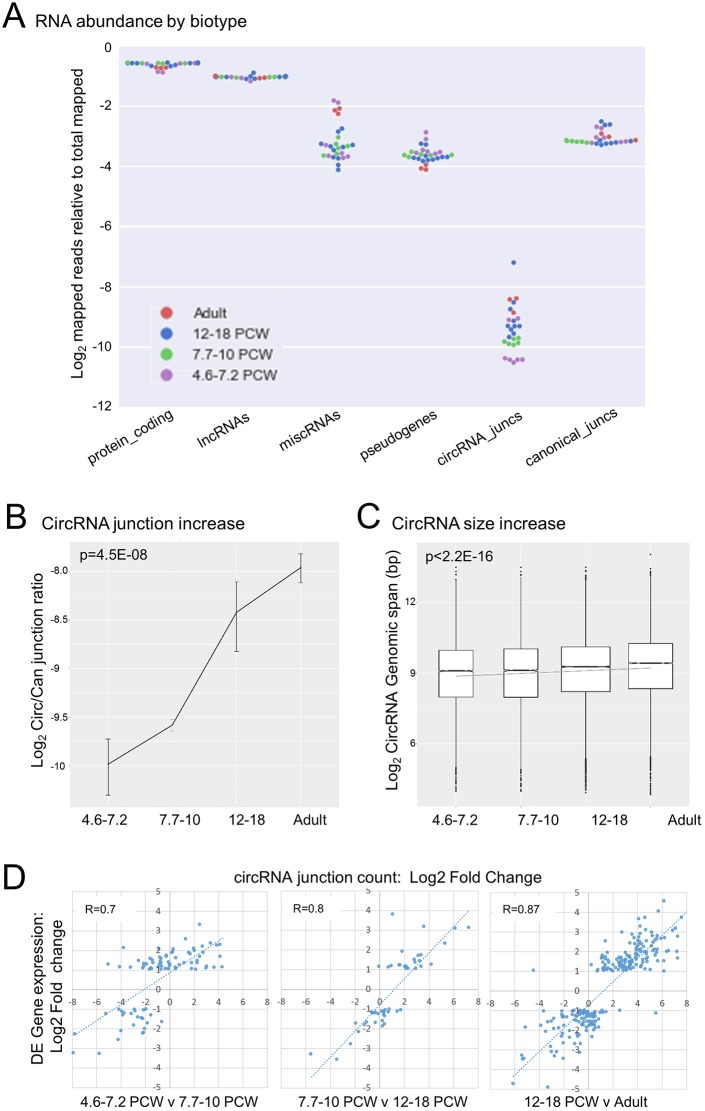
**circRNAs abundance increases during human retinal development.** (A) Swarm plot showing frequency of reads spanning circRNA (backsplice) junctions and canonical junctions in each sample relative to reads mapping to other RNA biotypes, as a proportion of the total reads mapped to all biotypes. Samples are colour coded according to the developmental windows defined by ME-based cluster analysis. (B) CircRNA enrichment across the developmental windows defined by ME-based cluster analysis. Ratios were derived by dividing total number of backsplice reads with canonical junction reads. Data are mean±s.e.m. The increase in number across all stages is statistically significant (Jonckheere-Terpstra test). (C) Boxplot showing distribution of circRNA sizes (genomic span between donor and acceptor splice sites) across the developmental windows defined by ME-based cluster analysis. The increase in size is statistically significant (Jonckheere-Terpstra test). Boxes define upper and lower quartiles, with the median indicated and outliers shown as solid circles. (D) Changes in abundance of circRNA derived from genes differentially expressed between developmental windows defined by ME-based cluster analysis. Pearson correlation coefficients are shown. Genes with very low circRNA expression levels at both time points being compared (<1 junction read per million reads per sample) were excluded from the analysis.

Non-coding RNAs are RNA molecules that are not translated into proteins. There are various types of non-coding RNAs and these include transfer RNAs (tRNAs), ribosomal RNAs (rRNA), piRNAs, siRNAs, lncRNAs and miRNAs. In the past 10 years, non-coding RNAs have been implicated in a variety of biological processes (Maiorano and Hindges, 2012). The abundance of various non-coding RNAs was assessed in all developmental windows defined through the Moran-Eigen vectors (Fig. 8A). Of various non-coding RNAs, circular RNAs (circRNAs) were the only group that showed an increase in abundance as development proceeded; hence, we performed a more detailed analysis of this RNA biotype.

CircRNAs (reviewed by Maiorano and Hindges, 2012) are a predominantly non-coding class of small RNAs, formed by ‘back-splicing’ events, the frequency of which can be affected by intronic homology (Jeck et al., 2013; Kramer et al., 2015), RNA editing (Rybak-Wolf et al., 2015; Aktas et al., 2017), RNA-binding proteins (Kramer et al., 2015; Ashwal-Fluss et al., 2014) and splicing factor availability (Liang et al., 2017). They have much longer half-lives than linear transcripts (Enuka et al., 2016; Zhang et al., 2016), and although most are expressed at low levels, some accumulate to become more abundant than linear transcripts from the same loci in many cell types (Guo et al., 2014). Although the vast majority have no known function, some, such as *CDR1-AS*/*CiRS-7* and *SRY* (Memczak et al., 2013; Hansen et al., 2013), act as microRNA sponges, others can enhance transcription by interacting with PolII (Li et al., 2015) and there is evidence that a small number undergo cap-independent translation (Pamudurti et al., 2017; Legnini et al., 2017). Notably, circRNAs are highly abundant in brain relative to other nucleated cell types, and can have dynamic spatiotemporal expression patterns (Barrett and Salzman, 2016; Szabo et al., 2015), leading to suggestions that they may play major roles in brain development during neural cell progenitor proliferation, differentiation and synaptogenesis (Venø et al., 2015; Xie et al., 2017).

To investigate changes in the circRNA population during retinal development, we first used PTESFinder (Izuogu et al., 2016), a tool with high specificity and sensitivity (Zeng et al., 2017), to identify all back-splice junctions within the dataset. A total of 36,244 distinct circRNA junctions were identified (Table S6). Although these were rare relative to canonical splices, representing less than 1% of all splice junctions in most samples, there was evidence of an increase in abundance during development, with samples from 4.6-7.2 PCW clustering separately (Fig. 8A). This increase was found to be statistically significant when ratios of circular to canonical junctions were grouped by developmental window (Fig. 8B, *P*=4.5E-08, Jonckheere-Terpstra test). Similar temporal changes have been reported in other neuronal differentiation series (Rybak-Wolf et al., 2015; You et al., 2015) and a dramatic increase prior to day 45 has been observed in a human embryonic stem cell (ESC) model of retinal differentiation (Izuogu et al., 2018). A modest, but highly significant, increase in circRNA size was also observed, again consistent with the ESC retinal model (Fig. 8C, *P*<2.2E-16, Jonckheere-Terpstra test). We therefore compared the abundance of all circRNAs identified in the retinal series and ESC model, and observed a strong correlation (R=0.84, Fig. S11A). Highly expressed circRNAs in both series include *CDR1as* (CiRS-7), a microRNA sponge for miR-7 and miR-671 (Memczak et al., 2013), the loss of which impairs sensorimotor gating in mice (Hansen et al., 2013), and circ*HIPK3*, a sponge for multiple micro RNAs (including miR-124), which may be necessary for cell growth (Zheng et al., 2016). However, the most abundant was derived from *RMST*, a long non-coding RNA known to regulate neural stem cell differentiation through co-regulation of neurogenic transcription factors with SOX2 (Ng et al., 2013). Analysis of the frequency of *RMST* splice junctions inside and outside of the circRNA confirmed that it accounts for >90% of *RMST* expression in all samples, and is approximately ten times more abundant in all developmental windows than in adult tissue (Fig. S11B), consistent with its reported role in neural differentiation.

Dynamic and divergent changes in expression patterns between circRNAs and linear transcripts from the same loci have been reported in some differentiation series, suggesting they can be regulated separately (Szabo et al., 2015; Venø et al., 2015; Rybak-Wolf et al., 2015; You et al., 2015; Ng et al., 2013). However, after correction for the global increase in circRNA levels, and locus-specific changes in linear gene expression (see Materials and methods), only 32 circRNAs had statistically significant different junction counts between adjacent developmental windows, suggestive of locus-specific regulation. Of these, 30 increased in expression over time, consistent with the global pattern of change (Table S7, see Materials and methods). To investigate the relationship between linear and circRNA expression further, we analysed total circRNA output from the 4.5% of circRNA producing genes (*n*=319) that showed differential expression between adjacent developmental windows (Table S2). Changes in total gene expression and circRNA junction counts were strongly correlated in all three analyses (r=0.7-0.87, Fig. 8D), and support the conclusion from an ESC model (Izuogu et al., 2018) that temporal changes in circRNA levels are tightly linked to total transcriptional output from their loci of origin during retinal differentiation.

Finally, we used PTESFinder to identify circRNAs present within the RNAseq data of Woods et al. (2018), derived from postnatal mouse retina, and compared them with those reported here. Of 25,971 circRNAs identified in mouse, 7358 (∼28.3%) overlap with human retinal circRNAs, and 2414 (∼9.3%) are fully conserved with respect to splice junction use (Table S8). Although this is lower than reported elsewhere (Jeck et al., 2013; Guo et al., 2014; You et al., 2015), variables such as library size, suitability of the comparator dataset, and measures of conservation used, complicate comparison between studies. The results do, however, identify a reduced subset of conserved retinal circRNAs, which are likely to be enriched for those with biological function.

## DISCUSSION

Although embryonic studies are widespread in other mammalian organisms and these have contributed greatly to our knowledge, characterising the events that occur specifically during human development is crucial in order to identify the differences that exist between humans and other organisms, and to better understand the pathogenesis of many forms of human disease arising from mutations in genes implicated in early development (Wadman, 2015; Hayden, 2016). Establishing and maintaining collections of human developmental tissue for direct study requires significant time and resources, and is not a viable option for all (Ledford, 2017). In this study, we have provided the spatiotemporal protein expression of key retinal cell markers and RNA-seq analysis of embryonic eyes as well as human embryonic and foetal retinal specimens that were cross compared with adult human retina. Inclusion of the very early eye samples, the detailed molecular and immunohistological analysis, together with the splicing and circular RNA analysis bring new and novel dimensions to this study, which informs the very early patterning events that govern lens, cornea, RPE and retinal formation, and which has not been published previously.

Our combined molecular and immunohistological analysis defined three key developmental windows: (1) 4.6-7.2 PCW, which is characterised by retinal progenitor proliferation, RPE and lens emergence, and is associated with the upregulation of genes acting in signalling pathways (TGF/BMP, WNT) involved in eye and retinal development; (2) 7.7-10 PCW, which is characterised by the emergence of RGCs and initiation of transcriptional programmes that underlie the development of interneurons (horizontal and amacrine cells) as well as cone photoreceptors; and (3) 12-18 PCW, which is characterised by the sequential emergence of cone, amacrine, rod, bipolar and Müller glial cells. Our study provides the first systematic analysis of alternatively spliced transcripts during human retinal development and identifies developmental stage-specific transcripts with alternative splicing that affects the formation of photoreceptor-connecting cilia during embryonic and foetal development (7.7-18 PCW), RNA splicing (12-18 PCW), and histone modification in the adult retina. We believe that the splicing switches that affect different sets of genes during retinal histogenesis can be used to predict the maturation stage of retinal cells generated from *in vitro* pluripotent stem cell differentiation, as highlighted in a recently published paper in mouse brain from Weyn-Vanhentenryck et al. (2018).

Finally, our analysis of the circular RNA population identified a transcriptome-wide increase in circRNA levels over time, broadly consistent with other longitudinal analyses of neuronal tissues across pre- and postnatal time periods (Szabo et al., 2015; Venø et al., 2015; Xie et al., 2017), although a reduction in circRNA abundance has been observed in some regions of the developing pig brain (Venø et al., 2015). Our analysis also identified circRNAs differentially expressed between developmental windows; however, among differentially expressed genes, circRNA levels were strongly correlated with changes in total gene expression.

Collectively, our data provide a resource of differentially expressed and alternatively spliced transcripts, circRNAs and protein expression during key stages of embryonic and foetal development, as well as in adult retinal tissue, which confirm and extend the available dataset currently available for the developing human retina (Hoshino et al., 2017; Tian et al., 2015). Many individuals affected by inherited disease lack a conclusive genetic diagnosis; screening of known associated genes will not always identify candidate variants as not all causative genes have been identified (Taylor et al., 2015). Tissue-specific gene isoforms are more frequently found in neural tissue (Liu and Zack, 2013; Cao et al., 2011); therefore, an accurate reference transcriptome of the human retina will help to identify the distribution of genetic variation and allow the identification of new pathogenic variants (Chen et al., 2015; Farkas et al., 2013). Furthermore, the elucidation of causal genes and their modifiers (Llavona et al., 2017) may help identify the underlying factors causing pathophysiology in these cases and allow novel therapeutic targets to be identified. Together, these data resources set the stage for benchmarking and improving pluripotent stem cell differentiation into retinal organoids, identifying new disease candidate genes and supporting the development of more effective therapies.

## MATERIALS AND METHODS

### Human tissue preparation

Human embryonic and foetal ocular material was dissected from foetal and embryonic terminations of pregnancies obtained from the MRC/Wellcome Trust-funded Human Developmental Biology Resource (HDBR, www.hdbr.org) with appropriate maternal written consent and approval from the Newcastle and North Tyneside NHS Health Authority Joint Ethics Committee. HDBR is regulated by the UK Human Tissue Authority (HTA; www.hta.gov.uk) and operates in accordance with the relevant HTA Codes of Practice. Samples that were 8 PCW or under were classified as belonging to a particular Carnegie stage using the embryo staging guides that can be viewed at http://www.hdbr.org/registration/staging.html, while foetal samples were staged using the criteria described in the fetal staging chart that can be downloaded at http://www.hdbr.org/registration/staging.html. Adult human retinal samples were collected with appropriate consent and approval from NRES Committee South East Coast (12/L0/0130). Developmental and adult tissue was collected into chilled Hanks balanced salt solution (HBSS) and transferred to a sterile petri dish containing fresh chilled HBSS for dissection. Tissue destined for RNA extraction was isolated, immediately immersed into RNALater (Ambion, AM7020) and stored at −20°C. Tissue for sectioning and immunostaining was fixed in 4% w/v paraformaldehyde (4% PFA) for at least 30 min (larger tissue was fixed for 1 h) then washed three times in phosphate-buffered saline (PBS) prior to processing. To isolate retinal and RPE tissues, eyes were secured in a cornea-side up position in a petri dish using fine forceps and a small incision made in the corneo-scleral junction with a small scalpel, through which the tip of curved micro-scissors was inserted. Eyes were carefully rotated 360°, and small incisions made all the way around the eye parallel to the corneo-scleral junction, to detach the anterior eyecup and lens from the posterior eyecup. The posterior eyecup was transferred to a small petri dish containing fresh chilled HBSS and the neural retina carefully separated from the underlying RPE using blunt dissection with fine forceps, then transferred into either RNALater or 4% PFA. The RPE was then blunt dissected away from the choroid using fine forceps and transferred into RNALater or 4% PFA.

### Immunohistochemistry

Tissue was fixed and IHC performed on cryostat sections as previously described (Mellough et al., 2015, 2012). Sections were reacted against a panel of retinal, lens and corneal-specific antibodies (listed in Table S9) that are widely used by many groups in retinal research worldwide. Antibody specificity was assessed by omitting the primary antibodies and testing all antibodies on adult human retinal tissue. Images were obtained using a Zeiss Axio Imager.Z1 microscope with ApoTome.2 accessory equipment and AxioVision or Zen software.

### Electron microscopy

For electron microscopy, tissue was fixed in 2% glutaraldehyde in 0.1 M sodium cacodylate buffer. For transmission EM (TEM) the tissue was post-fixed in osmium tetroxide, dehydrated in acetone and embedded in epoxy resin. Ultrathin sections (70 nm) were stained with uranyl acetate and lead citrate, and viewed on a CM100 TEM. For serial block face scanning EM (SBFSEM), tissue was incubated in a series of heavy metal solutions before being dehydrated and embedded in hard resin. The resin blocks were glued onto an aluminium pin and placed into a Zeiss Sigma SEM incorporating the Gatan 3view system, which allows sectioning of the block *in situ* and the collection of a series of images in the *z* direction. A region containing cone cells was imaged at ×1058 magnification (1750×3500 pixel scan), which gave a pixel resolution of ∼20 nm. Section thickness was 70 nm in the *z* direction. In the resulting *z* stack, the cone cells were segmented semi-manually using the watershed brush tool in Microscopy Image Browser (MIB, University of Helsinki, Finland). The segmentations were imported into Amira (FEI) for construction of the 3D model.

### RNA-seq

Once all the 32 eye and retina samples had been collected, RNA was extracted using the RNeasy Plus (QIAGEN). RNA quality was assessed using a 2100 Bioanalyser (Agilent) according to manufacturer's instructions. Samples with RIN>7.5 were processed for sequencing using the TruSeq Stranded Total RNA Library Prep Kit (Illumina) following manufacturer's instruction. Library quality and concentration was assessed using a Tapestation 4200 (Agilent Technologies) and a Qubit (Thermo Fisher Scientific). Libraries were pooled, to avoid any batch effects, and sequenced (∼50 million 75 bp paired-end reads per sample) on an Illumina NextSeq 500 (150 cycle High Output v2 kit). The base quality of the raw sequencing reads were checked using FastQC. The base quality of the raw sequencing reads were checked using FastQC. Trimmomatic (v0.32) was used to remove adapters and all reads shorter than 75 bp, and the last base at position 76. Reads were aligned to the Gencode GRch38, version 7, genome using STAR (v2.5.2b). The gtf file that was used was gencode.v25.chr_patch_hapl_scaff.annotation.gtf, which was downloaded from the Gencode website (https://www.gencodegenes.org/). After sorting and indexing the STAR produced bam files with samtools (v1.2), mapped reads were counted using htseq-count (v0.61). The average percentage of uniquely mapped reads was 90.05% (Table S1). All RNA-seq data have been deposited to GEO under accession number GSE98370.

To quantify the similarity of replicates within the same developmental stage, we computed the pairwise differences in expression of the same genes (i.e. across all samples from the same stage). This analysis confirmed that the expression levels were highly conserved across replicates. Representative distributions of the expression differences from embryonic (4.6 PCW), foetal (9 PCW) and adult stages, both in absolute and relative terms are shown in Fig. S12. The histograms of the absolute differences [|x-y|, where x=log(expression of a given gene in sample 1) and y=log(expression of the same gene in sample 2] demonstrate an exponential distribution, indicating that there is no bias in the expression across samples. The relative differences [|x-y|/(x+y+0.01)] show that the differences are proportionally small compared with the magnitude of the gene expression itself.

### Quantitative analysis

#### RNA-seq analysis

Read counts were imported into Rstudio (version 1.0.136) and normalized across samples using the ‘DESeq2’ package, which was also used for conducting the differential gene expression analysis. PCA plots were obtained using the built-in R function prcomp(). Gene annotation and search for human homolog genes of the mouse cell type markers were done using the ‘biomaRt’ Bioconductor package. The kurtosis of the gene expression distributions was computed using the ‘moments’ R package.

For the clustering analysis (Fig. 1), hierarchical clustering of all samples was performed using the hclust() function from the R package ‘stats’. This clustering was carried out using the ‘median’ agglomeration method and the pairwise gene expression distances as input. This distance matrix was obtained using the dist() function (also from the package ‘stats’), with the quantile-normalized [using the normalize.quantiles() function from the package ‘preprocessCore’] logarithm of the counts as input. The Moran Eigenvectors were computed from the resulting tree using the me.phylo() function of the ‘adephylo’ R package (Jombart et al., 2010). These vectors form an orthonormal basis, are centred to mean zero, with unit variance, and are not correlated with one another. Based on these Eigenvectors, the representation showing the samples' traits with respect to the Moran Eigenvectors was obtained using the table.phylo4d() function.

For the comparison of our dataset with the transcript counts dataset from Hoshino et al. (2017), log_2_-transformed counts per million (cpm) values were computed using the ‘edgeR’ package. The removeBatchEffect() function from the ‘limma’ R package was employed for these comparisons, prior to conducting the PCA analysis or the heatmap visualizations using the ‘pheatmap’ R package.

For the prediction of transcription factors (TFs), the cytoscape (Shannon et al., 2003; Verfaillie et al., 2014) plug-in iRegulon (Verfaillie et al., 2014) was used. iRegulon enables sequence-based discovery of regulons using motif discovery in a set of co-regulated genes. Approximately 200 of the top differentially expressed genes (among the four clusters identified in Fig. 1) were inputted to iRegulon (version 1.3, build ID 1024) using HGNC symbols. The ‘Predict regulators and targets’ functionality of iRegulon using the prespecified standard parameter set generated a list of the most prioritized predicted TFs (Table S3). These TFs are ranked according to their maximal normalised enrichment scores (NES), which quantify the extent to which the identified motif recovers an associated set of input genes.

#### Alternative splicing

This analysis was carried out using rMATS (Shen et al., 2014). For each comparison being made, we used the sorted BAM files produced by STAR to run rMATS using default unpaired settings. Reported splicing changes were considered significant if they had an adjusted *P*<0.05 and a change in inclusion level difference of more than 5%. GO Enrichment Analysis was carried out on the genes found to have significant splicing changes via clusterProfiler (Yu et al., 2012). Multiple testing corrections were carried out using the Benjamini-Hochberg method with an adjusted *P*<0.05 denoting significantly enriched gene ontology. We also carried out Biological Theme Comparison using ClusterProfiler Compare Cluster Function to reveal similarities in over-representation between our data and those published by Hoshino et al. (2017).

#### circRNA identification and analysis

Back-splice junctions were identified within merged paired-end reads using PTESFinder v1 [Izuogu et al., 2016 (parameters: JSpan=10, PID=0.85, segment_size=65], guided by splice junction annotations from Ensembl release 90 (Zerbino et al., 2018). To allow comparison with other biotypes, RNAseq reads were mapped to the human genome (GRCh38) using STAR v2.5.3a (Dobin et al., 2013) (parameters: --outFilterMultimapNmax 10, --outFilterMismatchNmax 2, --alignIntronMax 100,000). Reads mapping to Ensembl transcripts (release 90 were then extracted using BEDTools v2.26.0 Quinlan and Hall, 2010) and grouped by transcript biotype. Statistical analyses of trends in circRNA sizes and enrichment across sample groups were performed using the Jonckheere-Terpstra test for ordered differences, with the alternative hypothesis of increase over time. CircRNA and canonical junction counts from individual genes were normalised relative to library size. Differential expression analysis to identify circRNAs regulated independently of linear transcripts was performed using the method of Izuogu et al. (2018), which controls both for sample level differences in circRNA levels and locus level differences in total gene expression. Identification of conserved circRNAs was performed by using the UCSC LiftOver tool (Kent et al., 2002) to compare mouse retinal circRNAs (Westholm et al., 2014) with human (GRCm38 v GRCh38), and define those with either overlapping or precisely matching genomic coordinates.

## Supplementary Material

Supplementary information
