# Supplementary material for: An integrated transcriptional analysis of the developing human retina
Source: Development. 2019 Jan 29;146(2):dev169474. doi: 10.1242/dev.169474 (PMC6361134; doi:10.1242/dev.169474)
Supplement: Supplementary information [file develop-146-169474-s1.pdf]

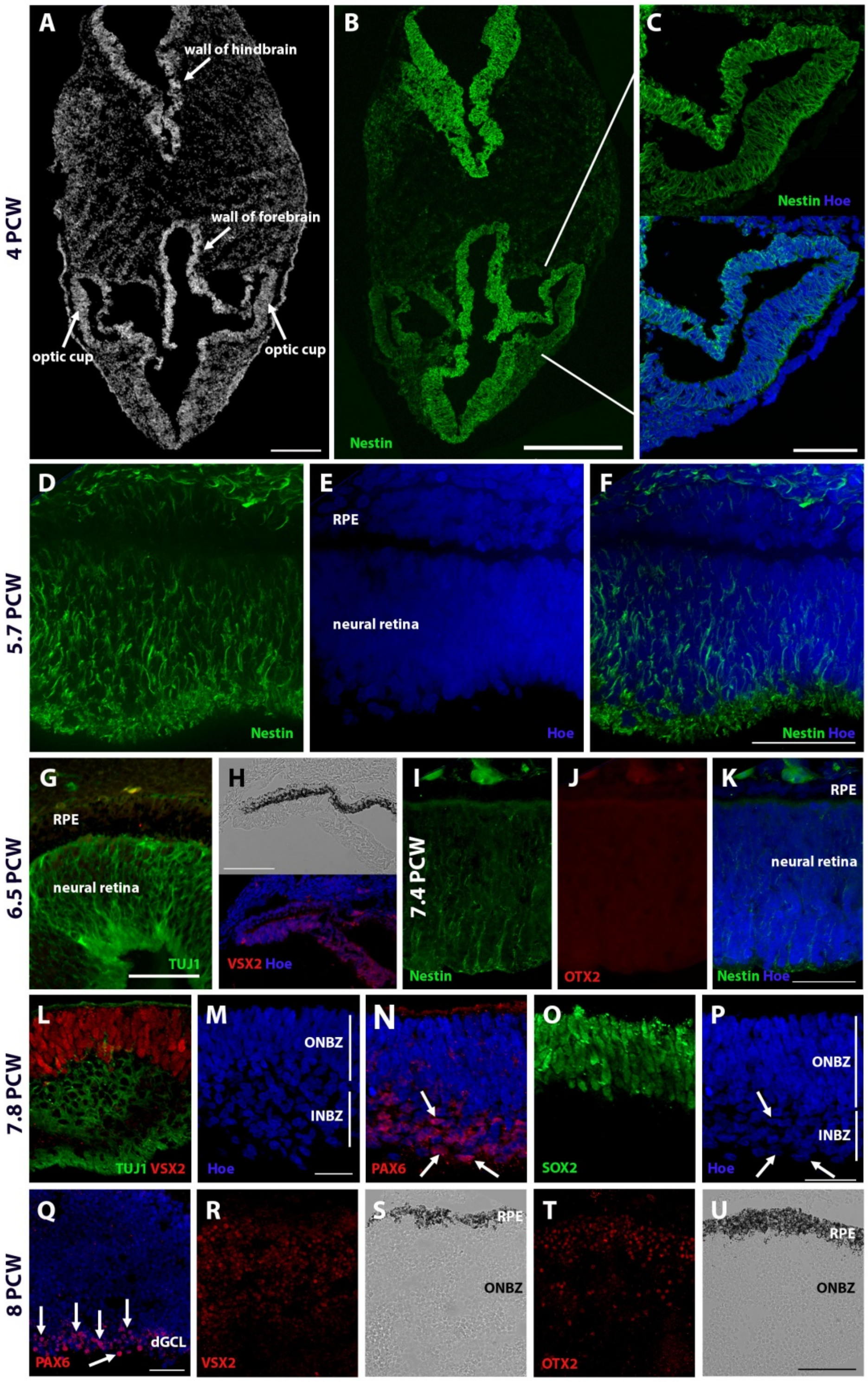

Figure S1. IHC analysis of sections through human embryonic brain and optic cups from 4 to 8 PCW. (**A-C**) A horizontal section through the embryonic brain at 4 PCW showing the bilateral appearance

Development • Supplementary information

of the developing optic cup, just prior to invagination. The optic cup and walls of the fore- and hindbrain are strongly immunopositive for the neural progenitor marker nestin. Sagittal sections through the optic cup showing the expression of TUJ1 (**G, L**), VSX2 (**H, L, R**), nestin (**D-F, I, K**), OTX2 (**J, T**), PAX6 (**N, Q**), and SOX2 (**O**) from 5.7 – 8 PCW. (C, E, F, H, K, M, P, Q) Hoechst staining is shown in blue. (H, S, U) bright phase microphotographs showing RPE. (Q, N) White arrows indicate PAX6 positive cells. Scale bars: A, B = 200µm, C, D, F, H, U = 100µm, G, K, P, Q = 50µm.

Development • Supplementary information

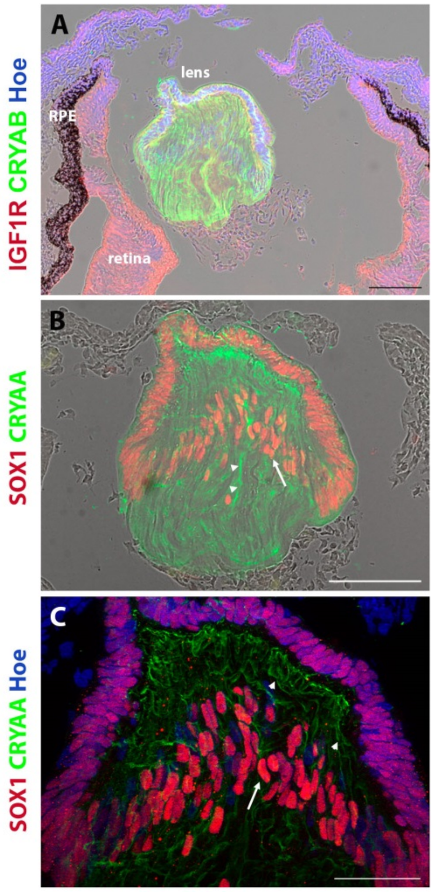

Figure S2. IHC expression analysis with IGF1R and lens markers CRYAB (**A**) and SOX1 and CRYAA at 6.3 PCW (**B**, **C**). Lens fibers are depicted by arrowheads and nuclei comprising the lens bow with arrows. Scale bars: A, B = 100µm, C = 50µm.

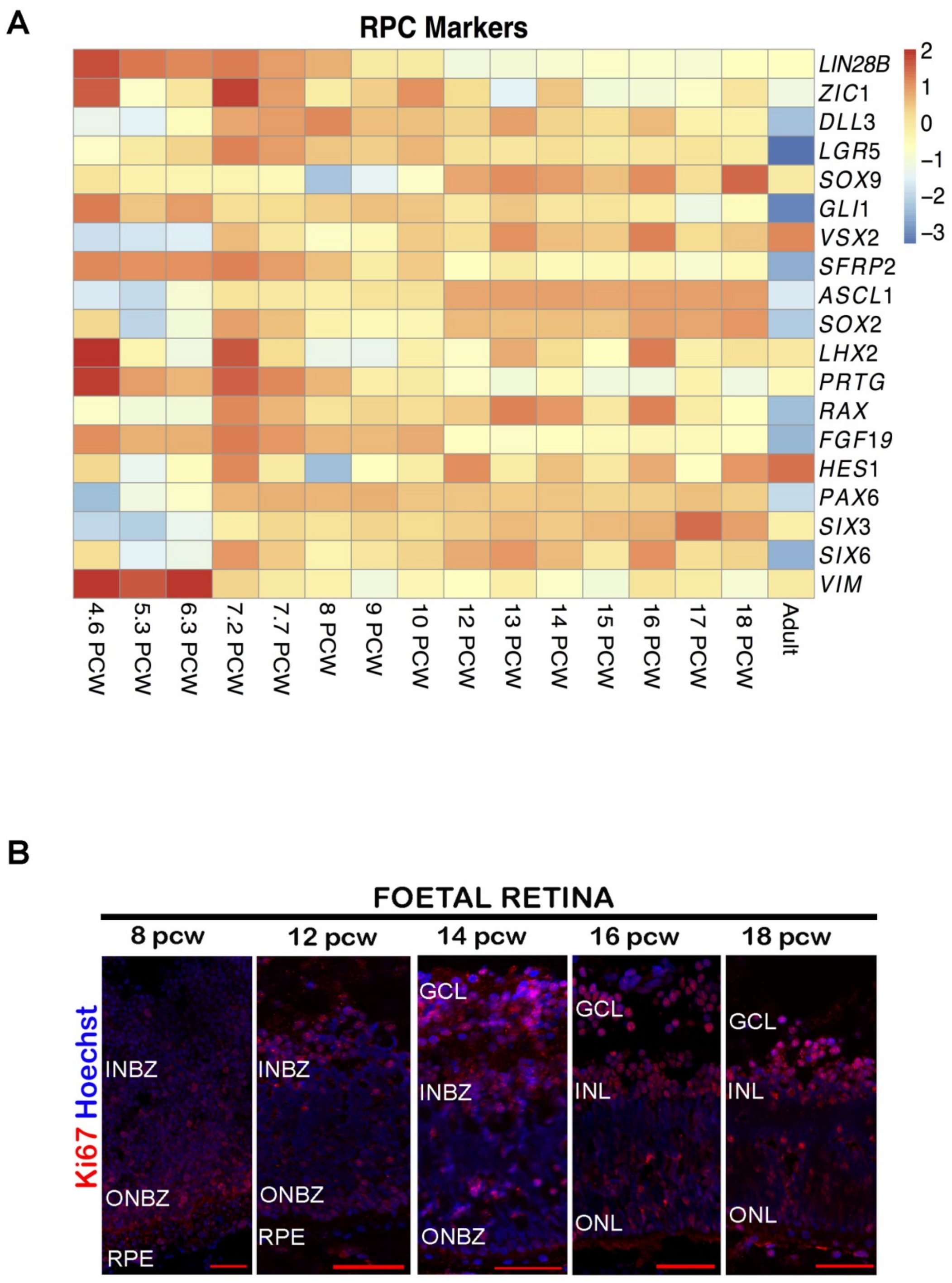

Figure S3. Expression of RPC markers during human retinogenesis. (**A**) Heatmap gene expression with RPC markers plotted as log2transformed counts per million (cpm) at each developmental stage. Blue to red represents low to high gene expression. (**B**) Ki67 immunostaining from 8-18 PCW of human retinal development. Scale bars 50 µm.

Development • Supplementary information

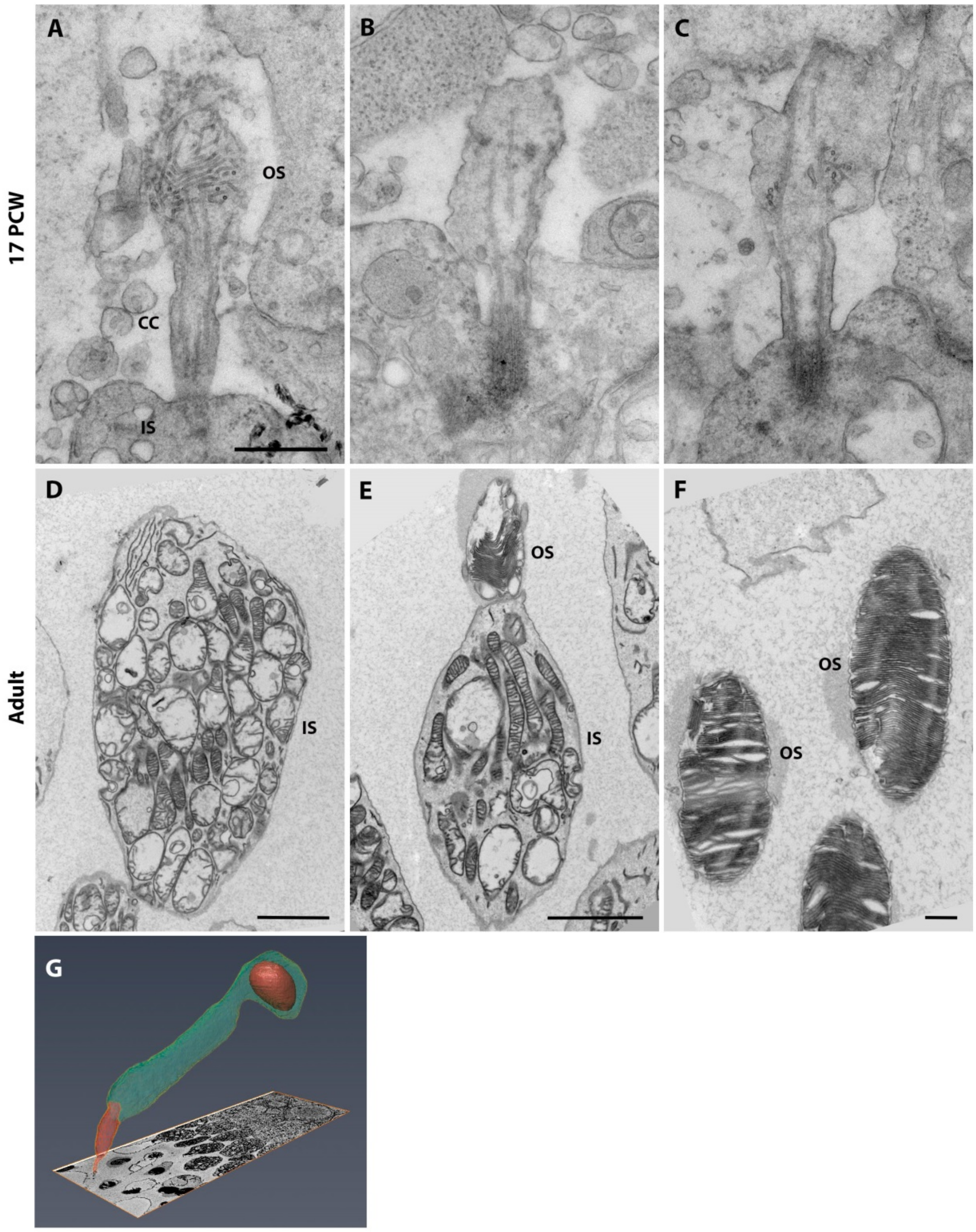

Figure S4. TEM analysis of a fetal retina at PCW17 (**A-C**) and adult retina (**D-F**). (**G**) 3D reconstruction of a cone photoreceptor from adult retina. OS - outer segments, cc- connecting cilia, is – inner segments. Scale bars: A-C, F = 500nm, D, E = 2µm.

Development • Supplementary information

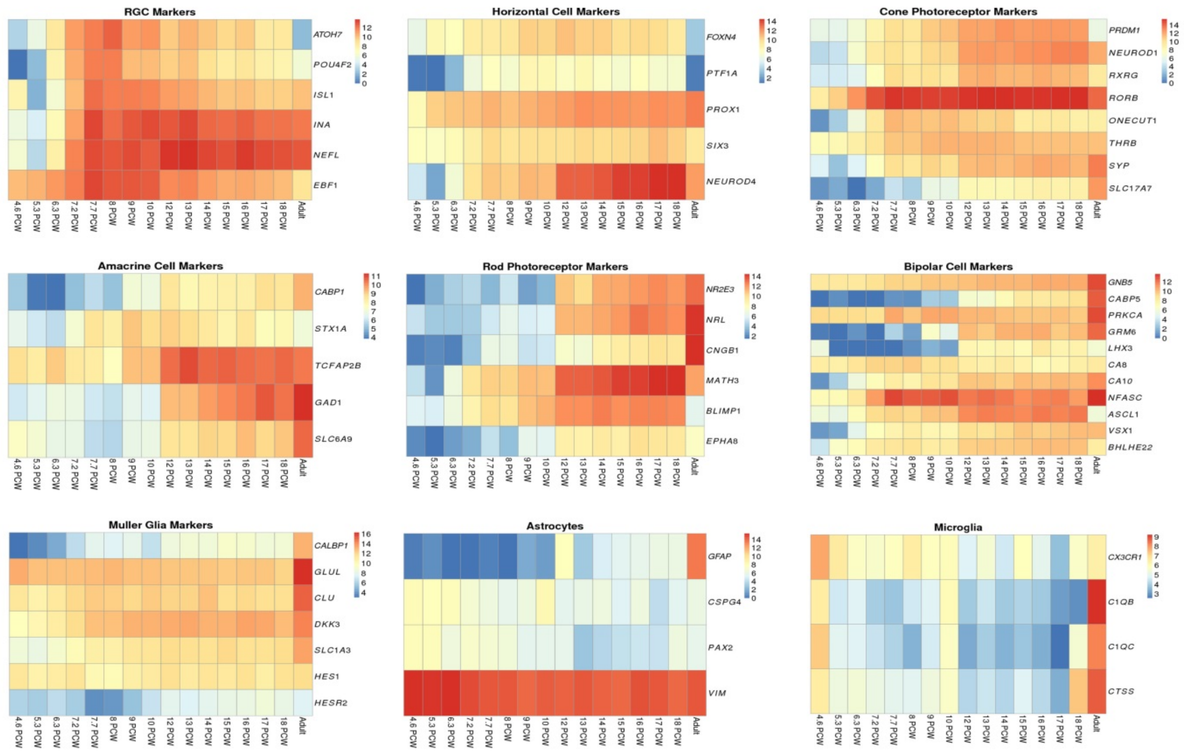

Figure S5. Heatmap gene expression with retinal cell markers plotted as log2transformed counts per million (cpm) at each developmental stage. Blue to red represents low to high gene expression.

Development • Supplementary information

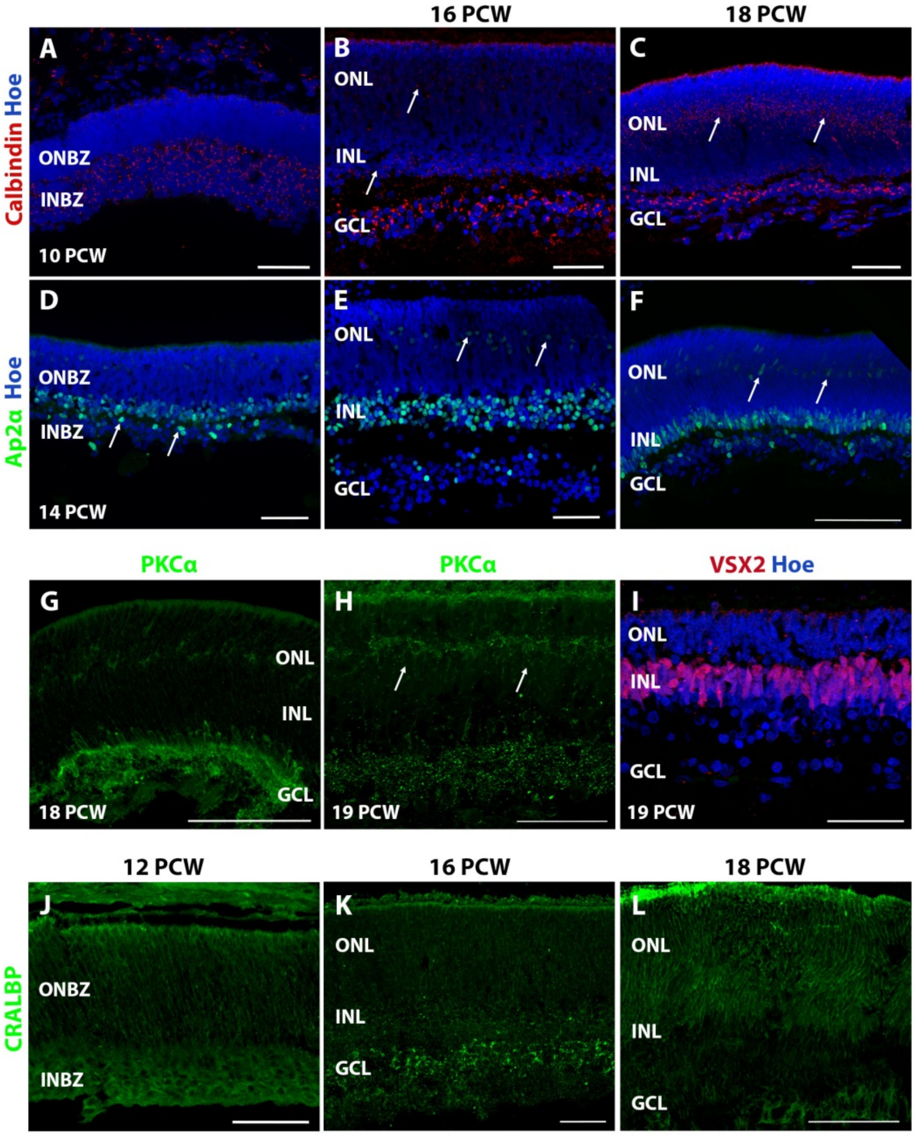

Figure S6. (A) IHC expression analysis with markers for amacrine and horizontal cells (**A-F**), bipolar cells (**G-I**), and Müller glia cells (**J-L**). Scale bars: A-E, H, I, J-L= 50µm, F, G, 100µm.

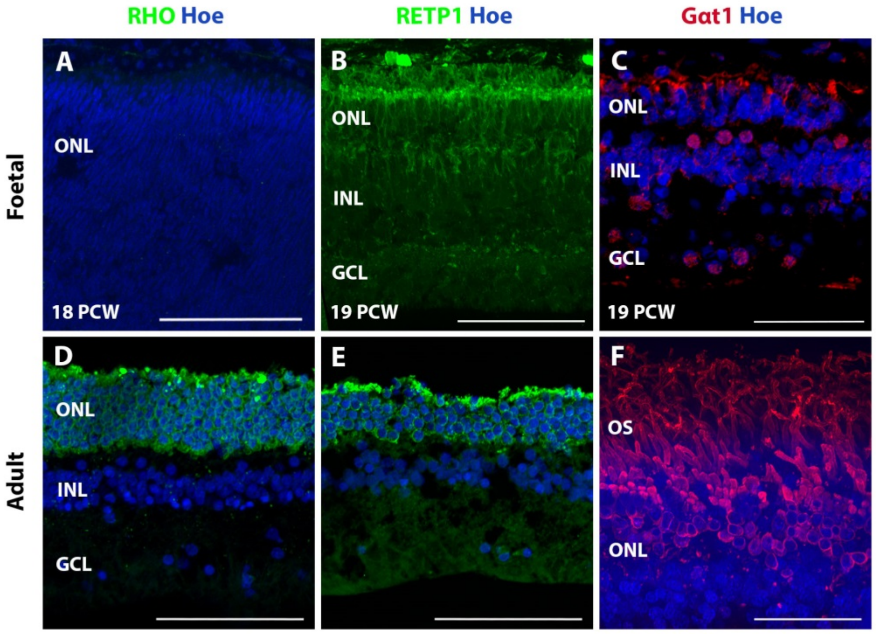

Figure S7. IHC expression analysis with rod markers (RHO, RETP1, GαT1 in fetal retina (**A-C**) and adult retina (**D-F**). Scale bars: C, F = 50µm, A, B, D, E = 100µm.

Development • Supplementary information

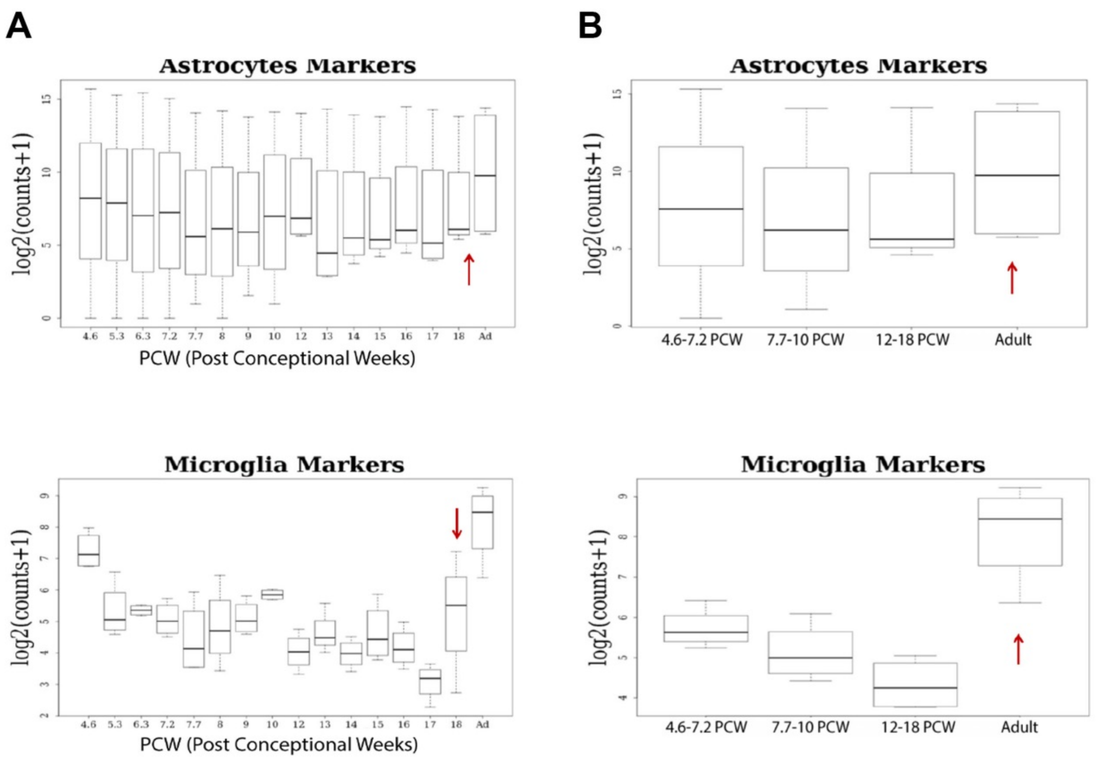

Figure S8. **The expression of astrocyte and microglia genes. (A)** Expression of marker genes was plotted as log2transformed counts at each developmental stage included in the RNA-seq analysis. The Wilcoxon rank-sum test was performed on the expression differences between developmental stages to identify ***the earliest stage*** with a significant and sustained increase in the expression of markers. Ad=adult retina; (**B**) Expression of markers across the three developmental windows (defined by ME-based cluster analysis) and adult retina plotted as log2transformed counts per million. The Wilcoxon rank-sum test was performed on the expression differences to identify developmental stages with ***peak expression*** of these markers. The red arrows indicate a significant and sustained increase in expression ($p < 0.05$).

Development • Supplementary information

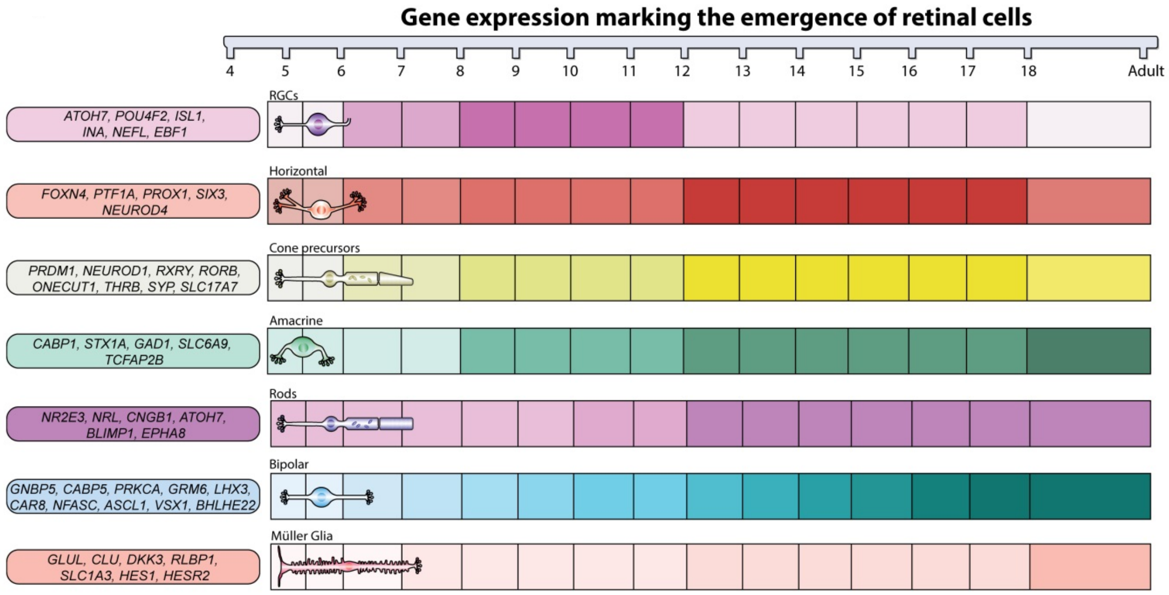

Figure S9. Schematic representation of retinal lineage marker expression during human retinal development. Increased colour intensity corresponds to an increase in marker expression.

Development • Supplementary information

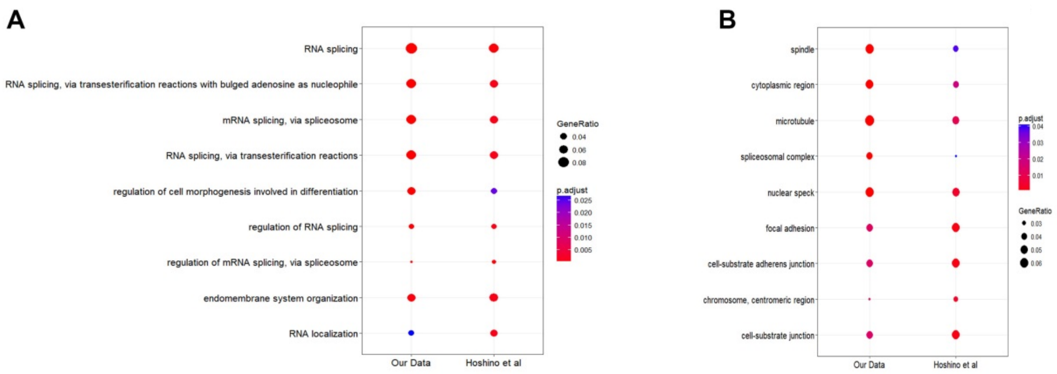

Figure S10. Biological Theme Comparison using ClusterProfiler Compare Cluster Function reveals similarities in over-representation between our data and those published by Hoshino et al [7]. Biological processes and cellular component analysis are shown in A and B respectively.

Development • Supplementary information

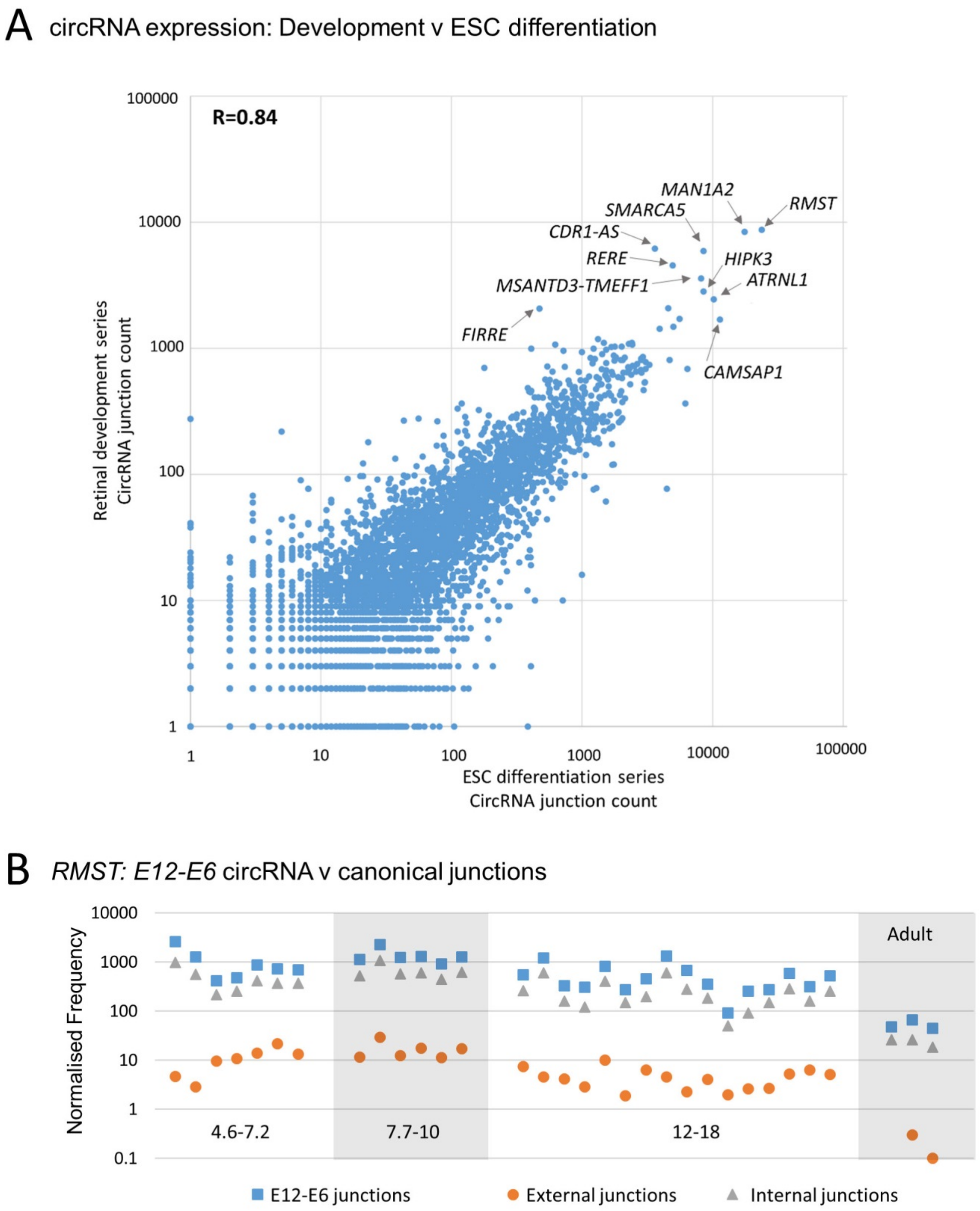

Figure S11. (**A**) Abundance of circRNAs relative to ESC retinal differentiation series (GEO data series GSE89957, [87]). Total circRNA junction counts from both series normalised to library sizes are shown, with origins of the most abundant circRNAs highlighted. *CDR1as* was not identified in the original ESC analysis, as the junctions are not within the GENCODE v19 reference set used. *FIRRE* is highlighted as it was the 2nd most abundant non-coding circRNA identified within ESCs, but is only expressed at high levels during PCW4.2-7.2. The Pearson correlation coefficient is shown. (**B**) *RMST* circRNA expression in developmental windows defined by the ME-based cluster analysis. CircRNA

Development • Supplementary information

junction counts from the dominant *RMST* circRNA isoform (E12-E6, chr12:97492460-97561047) are shown together with the average counts of canonical junctions internal to this circRNA (Internal: E6-E7, E7-E8…E11-E12), and the average counts of canonical junctions external to each circle (External: E1-E2,….E5-E6, E12-E13). All counts were normalised to library size.

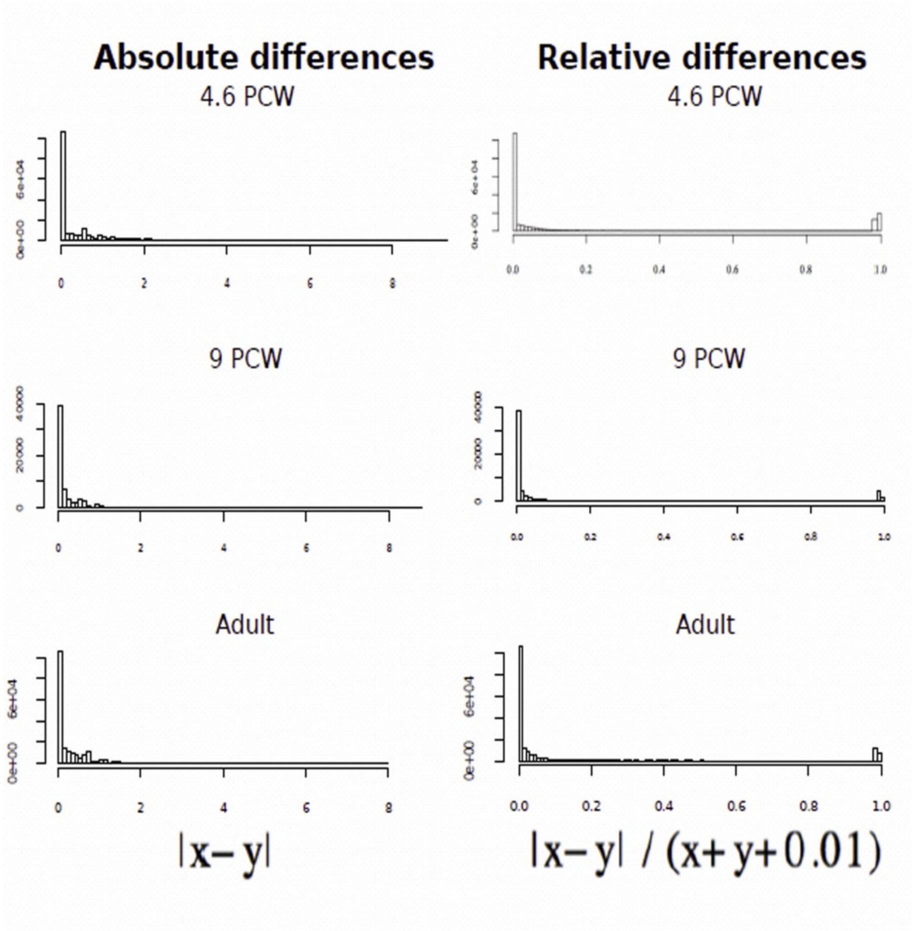

Figure S12. Absolute (left) and relative (right) pairwise differences of the expression of same genes across samples of the same developmental/adult stage. The expression is indicated as the logarithm of the counts. The exponential distributions are in accordance with the assumption that there is no bias in the expression between samples of the same developmental/adult stage. The relative distributions demonstrate that the counts are predominantly of the same magnitude.

Development • Supplementary information

Table S1. Summary of human embryonic and fetal specimens used for the RNA-seq analysis together with information of uniquely mapped reads.

Age (PCW)	Embryonic/fetal stage	Tissue	Number of input reads	Average input read length	Uniquely mapped read number	Uniquely mapped reads (%)	HDBR sample ID
4.6	CS14	Eye	48635815	149	31964221	65.7	N12301
4.6	CS14	Eye	45878621	149	35184170	76.6	N12354
4.6	CS14	Eye	83470237	149	73705339	88.3	N12878
5.3	CS16	Eye	72726041	149	66336156	91.2	N12556
6.3	CS18	Eye	75793100	149	69396960	91.5	N12675
6.3	CS18	Eye	70107419	149	63024290	89.9	N12612
7.2	CS20	Eye	71035011	149	62859161	88.4	N12527
7.7	CS22	Retina	81896361	149	74868307	91.4	N1897
7.7	CS22	Retina	74474840	149	68268223	91.6	N13187
8	CS23	Eye	90368108	149	83047597	91.9	N1901
9	F1	Retina	87114249	149	79908268	91.7	N13149
9	F1	Retina	79145487	149	72640814	91.7	N13159
10	F2	Retina	85520453	148	77759984	90.9	N12774
12	F4	Retina	77617400	148	70601893	90.9	N1940
13	F5	Retina	61158950	149	53513466	87.5	N12467
13	F5	Retina	80087667	148	72700299	90.7	N12915
14	F6	Retina	68108315	149	62752723	92.1	N13186
14	F6	Retina	73076639	149	64905340	88.8	N12552
14	F6	Retina	77087068	149	69272806	89.8	N12xxx5
15	F7	Retina	70503380	149	56632387	80.3	N12773
15	F7	Retina	64396321	149	57233955	88.8	N12xxx1
15	F7	Retina	81788089	149	73792351	90.2	N12xxx2
15	F7	Retina	79118938	149	71430605	90.2	N12xxx3
16	F8	Retina	78346354	149	72039696	91.9	N13132
16	F8	Retina	60797993	149	52286174	86	N12410
16	F8	Retina	85627649	149	76922127	89.8	N12638
17	F9	Retina	68388074	149	63021906	92.1	17 PCW
17	F9	Retina	81083394	149	74308837	91.6	N12xxx4
18	F10	Retina	63936406	149	47216106	73.8	18 PCW
Adult	Adult	Retina	62386081	149	50806136	81.4	Adult
Adult	Adult	Retina	56574426	149	43224561	76.4	Adult
Adult	Adult	Retina	64500755	149	49681505	77	Adult

Table S2. Differential gene expression analysis for each stage of development defined by ME-based cluster analysis (comparisons performed between two sequential stages for example 4.6-7.2 PCW vs 7.7-10 PCW). Log2 fold change of >1 and < -1 and padj< 0.05 were used as thresholds. Summary of GO terms for upregulated and downregulated genes are shown below the list of differentially expressed genes.

Click here to download Table S2

Table S3. Summary of transcription factors identified by iRegulon for each developmental stage defined by ME-based cluster analysis and available data on animal models retrieved from MGI and retinal disease database (OMIM).

Click here to download Table S3

Table S4. Differential exon usage analysis for each stage of development defined by ME-based cluster analysis (comparisons performed between two sequential stages for example 4.6-7.2 PCW vs 7.7-10 PCW). Inclusion difference >than 5% padj< 0.05 were used as thresholds.

Click here to download Table S4

Development • Supplementary information

Table S5. Summary of GO terms for biological process of differentially spliced transcripts for each stage of development defined by ME-based cluster analysis (comparisons performed between two sequential stages for example 4.6-7.2 PCW vs 7.7-10 PCW).

Click here to download Table S5

Table S6. CircRNAs identified in all samples. Splice donor and acceptor positions, and frequency, of all back-splice junctions are given. For every back-splice, the frequencies of all canonical junctions from the transcript annotation are also given

Click here to download Table S6

Table S7. Normalised total circRNA junction counts per gene compared between adjacent developmental windows, defined by ME-based cluster analysis. The student t-test was used to calculate p-values, with a Benjamini-Hochberg false discovery rate of 0.05 applied (padj).

Click here to download Table S7

Table S8. Human circRNAs conserved between human retina (this study) and mouse (Ref 88). Human genomic co-ordinates (GRChg38) are shown for each circRNA, together with ENSEMBL transcript identifier, donor and acceptor exons, gene name, and level of conservation (none, overlapping, or precise).

Click here to download Table S8

Development • Supplementary information

Table S9. Summary of antibodies used for IHC staining.

**Antibody Name**	**Catalogue Number**	**Source**	**Dilution**
Acetylated tubulin	T6793	Sigma	1:1000
AP2α	Sc-12726	Santa Cruz	1:200
CRALBP	GTX15051	Genetex	1:100
Crx (MO2) clone 4G11	H00001406-M02	Abnova	1:200
CRYAA		Gift from Roy Quinlan	1:50
CRYAB		Gift from Roy Quinlan	1:5
Gαt1 (K-20)	Sc-389	Santa Cruz	1:200
GFAP	Z0334	Dako	1:100
HuCD	A21271	Invitrogen	1:100
Islet 1/2	Sc-30200	Santa Cruz	1:200
Nrl	Sc-374277	Santa Cruz	1:100
Opsin blue	AB5407	Millipore	1:200
Opsin red/green	AB5405	Millipore	1:200
OTX2	Ab114138	Abcam	1:200
Pax6	PRB-278P	Covance	1:200
PKCα	610107	BD Transduction Laboratories	1:200
RAX	ARP31926-P050	Aviva Systems Biology	1:200
Recoverin	AB5585	Millipore	1:1000
RetP1	O4886	Sigma	1:200
Rhodopsin	Ab59260	Abcam	1:100
Smi32	SMI-32R	Covance	1:100
SOX1	4194S	Cell Signalling	1:200
SOX2	MAB2018	R&D	1:200
VSX2	HPA003436	Sigma Atlas	1:200

Development • Supplementary information
